# Stimulated Brillouin scattering in micro/nanophotonic waveguides and resonators

**DOI:** 10.1515/nanoph-2024-0732

**Published:** 2025-03-18

**Authors:** Linhao Ren, Wenyu Wang, Kang Xu, Liying Zhu, Jun Wang, Lei Shi, Xinliang Zhang

**Affiliations:** School of Mathematics and Physics, China University of Geosciences, Wuhan 430074, China; Wuhan National Laboratory for Optoelectronics, Huazhong University of Science and Technology, Wuhan 430074, China; Optics Valley Laboratory, Wuhan 430074, China; Xidian University, Xi’an 710126, China

**Keywords:** stimulated Brillouin scattering, micro- and nanophotonic waveguides, optical resonators

## Abstract

With the ongoing advancement of micro- and nanofabrication techniques, there has been a notable revival of interest in the field of stimulated Brillouin scattering within micro- and nanoscale waveguide structures in recent years. A variety of micro- and nanophotonic devices with different functions have been designed and fabricated, including lasers, amplifiers, isolators, sensors, filters, delay lines, and memory devices. Here, we provide a comprehensive review of stimulated Brillouin scattering in micro/nanophotonic waveguides and resonators on various promising material platforms, covering several key aspects such as the generation mechanisms of Brillouin nonlinear interactions in different waveguide structures and material platforms, methods for enhancing Brillouin gain, and a range of typical applications. Concluding our review, we offer insights into prospective future directions for this field.

## 1 Introduction

Stimulated Brillouin scattering (SBS) is a process generated by coherent photon–phonon coupling [1], [2], [3], [4], [5], with a history spanning over one hundred years in the fields of laser physics [6] and nonlinear optics [5], [7]. Brillouin scattering is a third-order nonlinear optical effect [8], characterized by an intensity that significantly outperforms both Kerr and Raman interactions by several orders of magnitude [9]. Brillouin nonlinear interactions arise from the coupling between optical fields and phonons in the GHz frequency range, which endows them with several unique and useful properties. Historically, research on Brillouin scattering has predominantly concentrated on optical fibers for an extended period [10], [11]. Initially, SBS was considered a detrimental effect and was viewed as undesirable in certain applications [12], such as passive optical network (PON) transmission [13] and high-power fiber lasers/amplifiers [14], [15]. To mitigate the adverse effects of SBS, several techniques have been explored, including phase modulation [11], introducing controlled temperature or strain gradients [16], and optimizing the fiber geometry [17]. However, with further research, SBS has demonstrated significant potential in a wide range of fields, such as sensing, Brillouin lasers/amplifiers, and microwave photonics. Due to the relatively weak acousto-optic interaction in optical fibers, it is typically necessary to observe SBS over tens of meters of fiber length. The advent of photonic crystal fibers has changed this situation, as within these fibers, Brillouin gain can be enhanced due to the stronger photon–phonon interactions in the microstructured core [18], [19]. Additionally, high-quality-factor optical microcavities significantly amplify the interaction between light and matter [20], positioning them as an exemplary platform for SBS research. In 2012, Rakich et al. formulated a groundbreaking theory suggesting that Brillouin interactions can be significantly enhanced through radiation pressure exerted on the boundaries of micrometer-scale waveguides [21]. These demonstrations, together with the development of the micro- and nanofabrication technologies, have sparked a period of intense research interest in SBS across various material platforms and micro/nanoscale waveguide structures.

At present, SBS has been realized in micro- and nanophotonic structures based on a variety of material platforms, including silica, chalcogenide glass, silicon, silicon nitride, lithium niobate, III–V materials, and fluoride crystalline materials. Different photonic waveguide materials have their own unique physical properties (Table 1), making it difficult to distinguish superiority or inferiority. For example, although silica (SiO_2_) and fluoride are superior materials that can fabricate optical microcavities with exceptionally high-quality factors, facilitating the development of low-threshold SBS lasers [22], they face considerable challenges in achieving on-chip integration. Silicon (Si) is a very mature integration platform, but due to the elastic mismatch between the silicon waveguide core and the cladding, it cannot confine acoustic waves. Therefore, to achieve SBS in silicon, it is often necessary to fabricate suspended waveguide structures to prevent the leakage of acoustic waves [23], which increases the cost and difficulty of fabrication. III–V materials, like aluminum nitride (AlN) and AlGaN, have a broad transparency window and are a potential candidate for achieving SBS in the visible band. However, these material platforms are often not compatible with modern complementary metal-oxide-semiconductor (CMOS) nanofabrication facilities. Chalcogenide glasses, such as arsenic sulfide (As_2_S_3_) and GeSbS, possess high refractive indices and relatively low stiffness, enabling the confinement of both optical and elastic waves through total internal reflection. As a result, the first demonstration of SBS in an integrated photonic platform was achieved using chalcogenide glass [24]. Nonetheless, this material confronts a significant challenge: its low laser damage threshold. Silicon nitride (Si_3_N_4_) [25] and lithium niobate (LiNbO_3_) [26] have recently emerged as favorites in the field of SBS research due to their excellent performance in terms of propagation loss and nonlinear effects. However, a significant challenge remains in further enhancing the SBS gain coefficient. With such rapid development in the field, it is challenging to predict which material will become dominant. However, it is anticipated that for a considerable time in the future, SBS research will continue to span across various material platforms, and even hybrid platforms of multiple materials, in order to leverage the strengths of different materials.

**Table 1:** Key physical properties of photonic waveguide material platforms for SBS.

Material platform	Refractive index @ 1,550 nm	Photoelastic coefficient	Young modulus/GPa	Density/(kg/m^3^)	Thermo-optic coefficient/K^−1^
SiO_2_	1.44	*p*_11_ = 0.12, *p*_12_ = 0.27 [27]	73	2,200	6 × 10^−6^ [28]
As_2_S_3_	2.3 [29]	*p*_11_ = 0.308, *p*_12_ = 0.299 [30]	75.79	3,430	–
GeSbS	2.28 [31]	*p*_11_ ≈ *p*_12_ ≈ 0.238 [31]	31.9 [31]	4,300	3.1 × 10^−5^ [32]
Si	3.48	*p*_11_ = −0.094, *p*_12_ = 0.017 [33]	131	2,329	1.86 × 10^−4^ [34]
Si_3_N_4_	2	*p*_12_ = −0.047 [25]	310	3,100	2.9 × 10^−5^
AlN	2.12 (*n*_o_), 2.16 (*n*_e_) [33]	*p*_11_ = −0.1, *p*_12_ = −0.027 [33]	330	3,260	6 × 10^−5^ [35]
LiNbO_3_	2.21 (*n*_o_), 2.14 (*n*_e_) [33]	*p*_11_ = −0.026, *p*_12_ = 0.09 [33]	210	4,700	3.3 × 10^−5^
MgF_2_	1.37	*p*_11_ = 0.25, *p*_12_ = 0.27	120	3,100	–

Here, we provide a detailed summary of the research advancements in Brillouin scattering across various material platforms, as well as the various strategies employed to enhance Brillouin nonlinear interactions. Our focus is primarily on micro/nanoscale waveguide and resonant cavities, including micro/nanofibers, quasi-planar waveguide geometries, and microcavities. Additionally, we explore the potential applications of SBS across a range of fields, such as microlasers, microwave photonics, nonreciprocal transmission, sensing, and optical storage.

## 2 Fundamentals of stimulated Brillouin scattering

**Figure 1:**
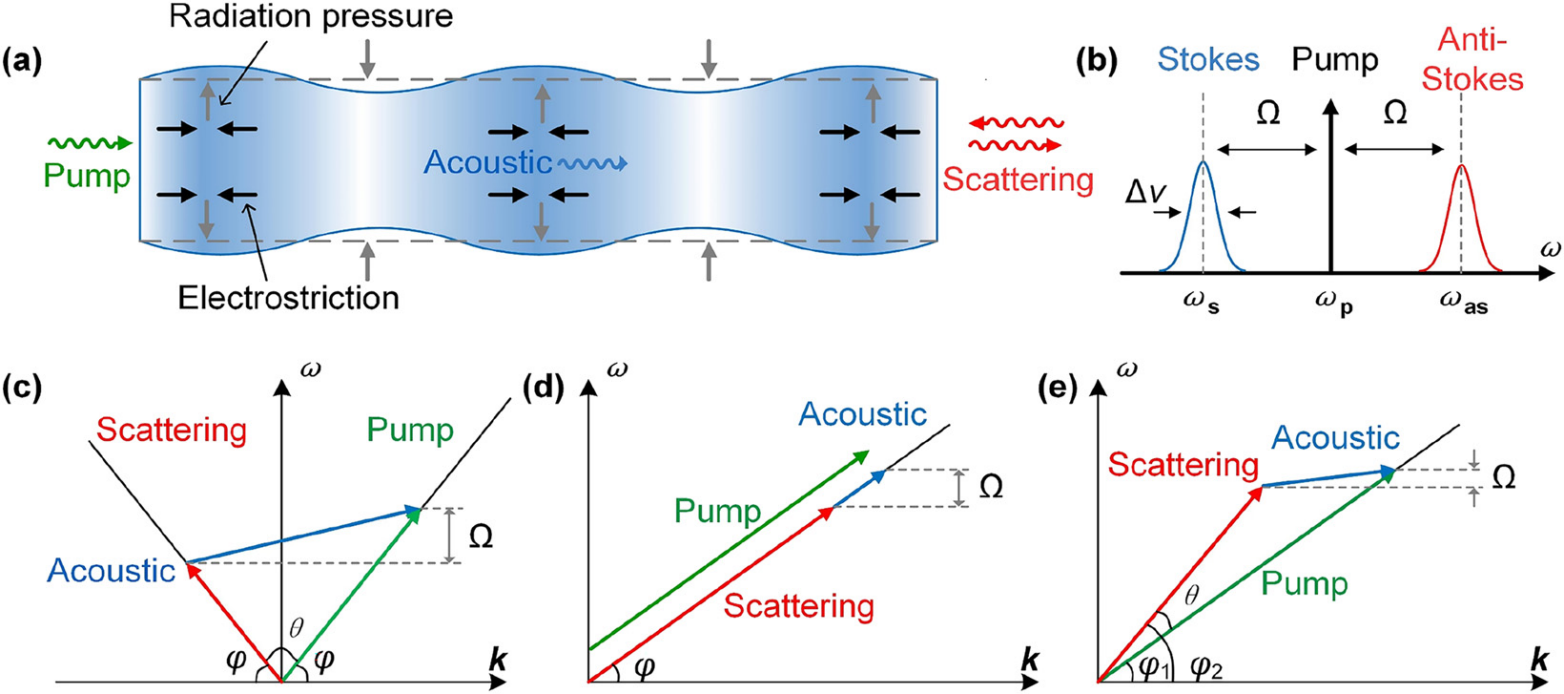
The basic principle of stimulated Brillouin scattering. (a) Waveguide refractive index change and surface deformation caused by electrostriction and electrostriction. (b) Stokes and anti-Stokes Brillouin scattering process. (c–e) Dispersion diagram for backward SBS, forward SBS, and intermodal SBS.

Brillouin scattering is a nonparametric process that involves the interaction between incident light and the energy levels of matter. The crystal lattice within the medium undergoes constant thermal motion, generating intrinsic acoustic waves. Spontaneous Brillouin scattering occurs when photons are scattered by these thermally excited acoustic phonons [36]. In contrast, SBS is a process where the interaction between optically excited acoustic waves and incident light forms a feedback loop to exponentially amplify the scattered light [37]. As shown in Figure 1a, the incident optical wave changes the material density of a waveguide or deforms its surface through electrostriction and radiation pressure, resulting in a light-excited acoustic wave. The acoustic wave periodically modulates the permittivity, forming a moving refractive index grating that scatters the incident pump light into the Stokes or anti-Stokes signal with a Brillouin frequency shift. In turn, the interference of the pump light and the scattered light enhances the electrostriction, further promoting the Brillouin scattering process [38]. As shown in Figure 1b, SBS is distinguished according to whether the phonon is emitted or annihilated. For the Stokes Brillouin transition, a high-frequency pump photon is converted into a lower-frequency Stokes photon, generating a phonon in the process. Conversely, in the anti-Stokes process, a lower-frequency pump photon is converted into a higher-frequency anti-Stokes photon, annihilating a phonon [39]. Both Stokes and anti-Stokes processes must observe the conservation of energy and momentum [9]:

(1) $$q=\left|k_{s,as}-k_{p}\right|\;\text{and}\;\Omega =\left|\omega_{s,as}-\omega_{p}\right|$$

where (*k*_*p*_, *ω*_*p*_) and (*k*_*s*_, *ω*_*s,as*_) are the wave vector and frequency of the pump light and the Stokes (anti-Stokes) light, respectively, while *q* and Ω denote the wave vector and frequency of the acoustic wave. Because the frequency of light wave is much higher than that of acoustic wave (*ω*_*s*_ ≈ *ω*_*p*_ >> Ω), Ω can be expressed as:

(2) $$\Omega =2V_{a}\cdot k_{p}\cdot \sin\frac{\theta }{2}$$

where *V*_*a*_ is the acoustic velocity in matter and *θ* is the intersection angle between the pump and scattered light. The frequency Ω is determined by the frequency of the pump light and material parameters of the waveguide, ranging from the hundreds of megahertz to tens of gigahertz. Brillouin gain *G*_*B*_ is used to describe the efficiency of SBS in different materials, which is given by [40]:

(3) $$G_{B}=\frac{g_{B}\cdot P\cdot L}{A_{\text{eff}}}$$

with *P* being the power of the pump light, *L* the interaction length, and *A*_eff_ the effective mode area. *g*_*B*_ is the Brillouin gain coefficient, which has a Lorentz spectral line distribution [41]:

(4) $$g_{B}=g_{0}\frac{{\left(\Delta v\right)}^{2}}{\left(\Omega -\Omega_{B}\right)+{\left(\Delta v\right)}^{2}}$$

This means that SBS has the maximum gain coefficient *g*_0_ when the acoustic wave frequency Ω = Ω_*B*_. The gain coefficient *g*_*B*_ can be calculated through a full-vectorial simulation of optical-acoustic coupling with the finite-element method [42]. The linewidth Δ*v* of the Brillouin gain spectrum is determined by the lifetime of the phonon mode with a typical value of tens to hundreds of megahertz for silica [43]. Such a narrow linewidth makes Brillouin scattering a popular technique for applications such as filtering or laser linewidth narrowing.

### 2.1 SBS in micro- and nanoscale optical waveguides

Micro- and nanoscale optical waveguides confine the light field to a very small mode volume. Therefore, it is easier to observe Brillouin scattering compared to bulk materials due to the stronger interaction between light and the matter. It can be concluded that there are generally three types of SBS in waveguides from the equation (2), namely, backward Brillouin scattering, forward Brillouin scattering, and intermodal Brillouin scattering [27] as shown in Figure 1c–e. When single-mode waveguides are considered, there are only forward (*θ* = 0) and backward (*θ* = *π*) Brillouin scattering. Backward scattering has a much larger Brillouin frequency shift (normally ∼10 GHz) than forward scattering (∼10 MHz–1,000 MHz) [27], [43]. For backward Brillouin scattering, pump light and scattered light counterpropagate and exchange energy via an acoustic mode as shown in Figure 1c. Since the acoustic mode possesses a large wave vector along the waveguide direction, it is constrained in the core of the waveguide and cannot reach the waveguide boundary. Therefore, backward Brillouin scattering mainly relies on electrostriction to alter the local refractive index of the waveguide, rather than deforming the waveguide surface [8]. For forward Brillouin scattering, as shown in Figure 1d, the copropagating pump light and scattered light couple with each other through an acoustic mode with a wave vector that is close to, but not exactly equal to, zero. This means that almost no sound waves are transmitted along the waveguide. Such an acoustic mode has strong transverse components extending to the waveguide boundary and leaks into the substrate. Therefore, it is usually necessary to design special waveguide structures to restrict the forward Brillouin acoustic modes, such as suspended waveguides. This kind of scattering is also referred to as “guided acoustic wave Brillouin scattering” (GAWBS) [44]. The acoustic wave in forward Brillouin scattering is a surface wave, which deforms the surface of the waveguide by radiation pressure. Intermodal Brillouin scattering can only occur in multimode waveguides and involves the coupling between different-order optical modes, which have wave vector directions with different angles as shown in Figure 1e. Therefore, it can take place either in the forward or backward direction, and each case possesses properties similar to those of conventional forward or backward Brillouin scattering. However, since the pumped mode and the scattered mode have different spatial distributions, it is crucial that the acoustic mode possesses the correct field distribution to support the interaction [45].

Brillouin gain *G*_*B*_ is largely determined by the overlap between the optical and acoustic modes in the waveguide, which can be quantified by the mode field overlap coefficient. This coefficient describes how effectively the optical and acoustic fields interact within the waveguide structure. Different methods to achieve efficient Brillouin gain have been reported. One approach is to fabricate waveguides using materials with a high refractive index and low stiffness. The high refractive index localizes the light field into a smaller mode volume, and the low stiffness contributes to phonon excitation in the waveguide. Another approach is to mechanically isolate the optical waveguide core from the substrate, thereby confining the acoustic waves to the waveguide core. This is commonly used in forward Brillouin scattering because its large transverse component can extend to the waveguide boundary. These methods will strengthen the optical mode and acoustic mode constraints of the waveguide, thereby improving Brillouin gain. In addition, increasing the mode overlap factor between optical modes and acoustic modes also plays an important role in improving Brillouin gain, especially for intermodal Brillouin scattering, which involves different-order optical modes. SBS in micro- and nanoscale waveguides has similar properties to that in optical fibers, meaning many applications based on Brillouin scattering in fibers can also be directly implemented on micro- and nano-waveguide platforms.

### 2.2 SBS in optical resonators

**Figure 2:**
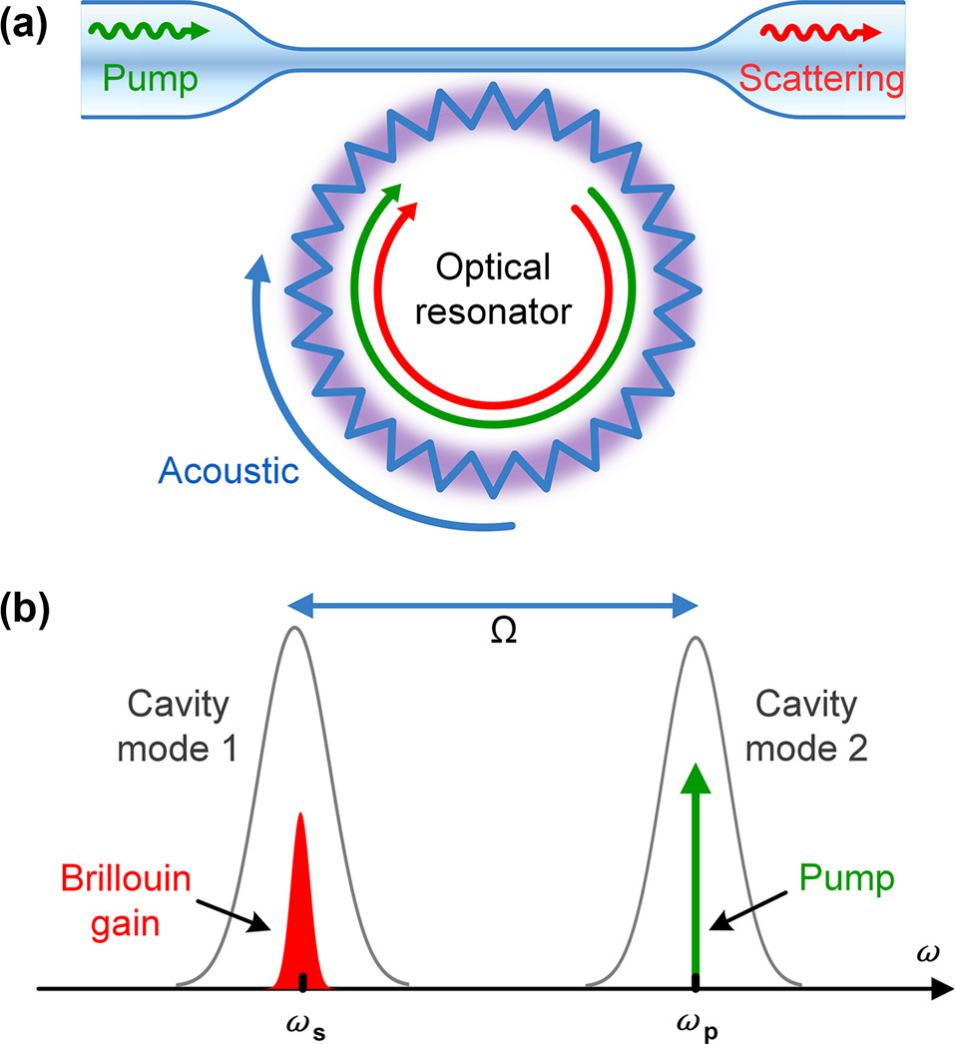
Stimulated Brillouin scattering in optical resonators. (a) Schematic illustration of the light–acoustic wave interaction in a WGM microcavity. (b) Pump light and Brillouin scattered light simultaneously resonance with different cavity modes.

As mentioned earlier, the Brillouin scattering gain *G*_*B*_ is proportional to the length of a waveguide. Therefore, a long waveguide with ultralow loss is usually required to achieve large Brillouin gain, which is essential for the SBS process. This hinders the development of integrated devices. An approach to solve this problem is utilizing the resonant mode of an optical cavity to enhance Brillouin interaction [46]. Whispering-gallery-mode (WGM) microcavities have been the excellent platform for researching low-power nonlinear optical phenomena during the last decade due to their ultrahigh quality factor (*Q*) and very small modal volume (*V*), which cause huge energy intensity and significantly enhance the interaction between light and matter inside the resonators [47], [48]. Therefore, it is useful to utilize a WGM microcavity to decrease the SBS threshold [49]. In order to satisfy the conservation of energy and momentum, there are some limitations on Brillouin scattering in WGM microcavities. As shown in Figure 2, the pump light and the Brillouin scattering light must simultaneously be in the different resonant modes of the microcavity, and the frequency difference of the resonant modes is equal to the phonon frequency of the material [50].

However, in order to match the phonon frequency to the free spectral range (FSR) of the microcavity, a submillimeter-sized WGM resonator is usually required [27], [51], [52]. Another approach to accomplish this is selecting two different-order WGMs (fundamental or higher order modes), which are close enough to address the pump and Stokes frequency. In this way, the Brillouin scattering process can be promoted by resonance. This means that the Brillouin scattering process in the microcavity usually belongs to intermodal Brillouin scattering, where the overlap between optical mode fields and acoustic mode fields plays an important role in Brillouin gain. When the gain is equal to the loss in the single round trip, the Brillouin laser emits and the threshold of SBS in a WGM microcavity can be given as [53]:

(5) $$P_{\text{th}}=\Gamma \cdot \frac{\pi^{2}n^{2}V_{p}}{g_{B}\lambda_{p}\lambda_{s}Q_{p}Q_{s}}\cdot \frac{1}{1+Q_{a}\lambda_{a}/2\pi R}$$

where *Q*_*i*_ and *λ*_*i*_ (*i* = *p*, *s*, *a*) are the *Q* factors and wavelengths of pump, Brillouin scattering and acoustic wave, and Г is the overlap factor of optical modes and acoustic modes. According to equation (5), the threshold of SBS is positively correlated with *V*_*p*_/*Q*_*p*_ *Q*_*s*_. Benefit from the ultrahigh *Q* factor and small *V*, there has achieved Brillouin lasing with a threshold as low as microwatt level in WGM microcavities [54]. Furthermore, the process can yield Brillouin laser emission that is orders of magnitude more spectrally pure than the pump light due to the narrow gain spectrum. The linewidth-narrowing property of Brillouin lasers is particularly promising as a means of generating laser sources and microwave sources [52], [55].

## 3 Silica

Silica material, especially in their glass form, is widely acknowledged for their outstanding optical properties, such as wide transparent window, low optical loss, high damage threshold, and outstanding thermal stability, which render it essential in diverse optical and photonic applications. Optical fibers possess distinctive elastic characteristics, rendering them an ideal material for study and utilization of Brillouin light scattering [11]. In 1972, the first observation of SBS in optical fibers was reported [10]. The low SBS threshold power was promptly exploited to demonstrate the first Brillouin laser with a fiber loop serving as a laser cavity [56]. The Brillouin effect in optical fibers was further employed to showcase the first semiconductor laser-pumped Brillouin fiber amplifier in 1987 by Olsson et al. [57]. Following decades of research, the SBS effect in optical fibers has been studied in detail. In recent years, the SBS effect in micro/nanofibers and WGM microcavities has garnered extensive attention. Both of them are capable of confining optical modes and enhancing the SBS gain. This review summarizes the recent advancements and applications of the SBS effect in silica micro/nanofibers and WGM microcavities.

### 3.1 SBS in silica micro- and nanofibers

**Figure 3:**
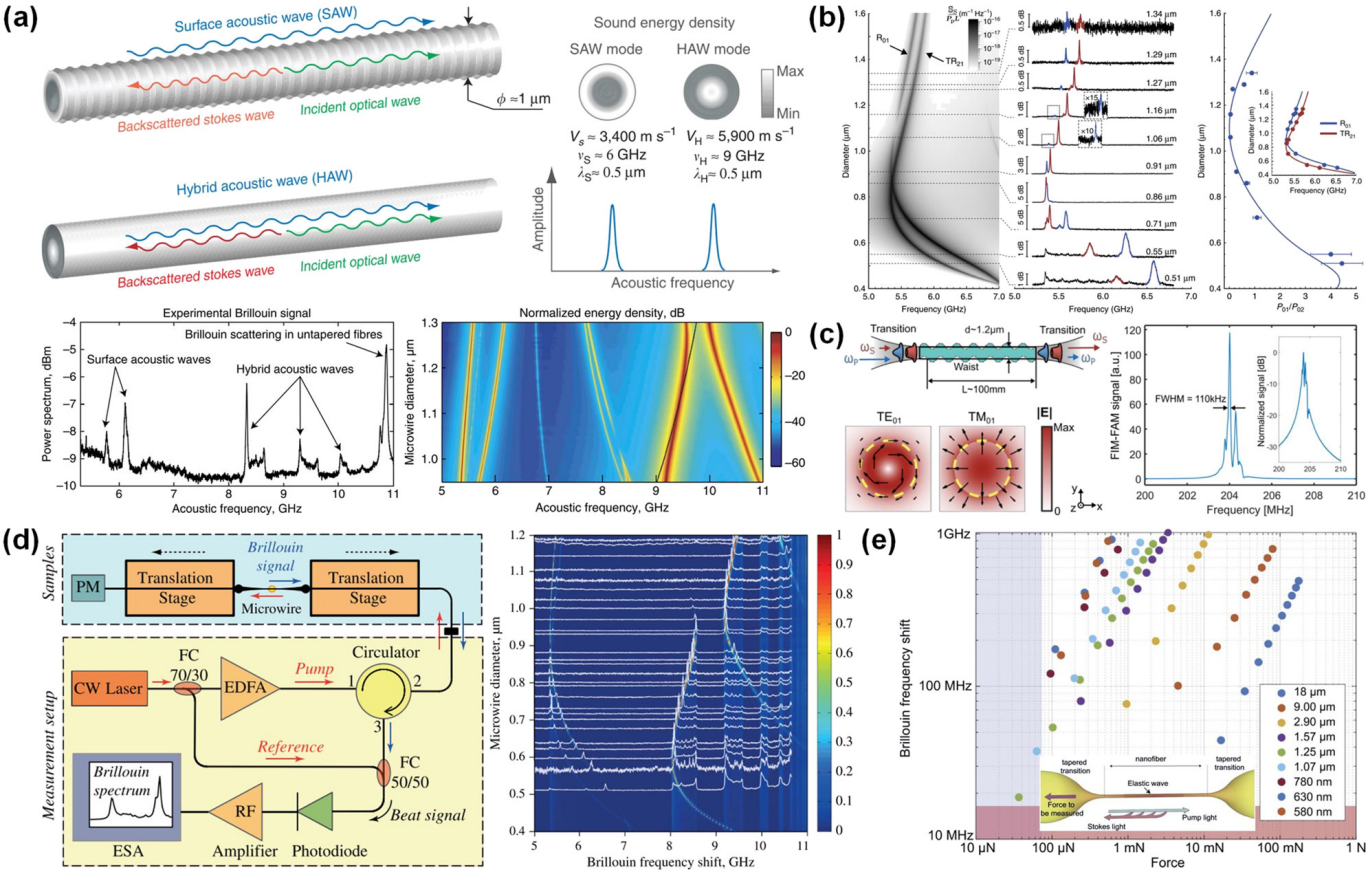
SBS in silica micro/nanofibers and its applications. (a) Upper: schematic representation of a silica microfiber and of the wavevector interaction for SAW and HAW. Lower: experimental Brillouin spectrum and numerical simulation of Brillouin scattering spectrum. Reproduced with permission [58]. Copyright 2014, Springer Nature. (b) Observation of the Brillouin scattering self-cancellation effect [61]. (c) Left: propagating flexural mode of a fiber taper driven by phase-matched pump and Stokes optical beams. Right: TE_01_ Brillouin resonance [62]. (d) Fiber taper diameter characterization [63]. Copyright 2017, Optica Publishing Group. (e) Force sensing [64].

Silica micro- and nanofibers exhibit unique mechanical, acoustic, and optical properties, thus being of significant importance in numerous research fields. These tapered subwavelength waveguides are capable of confining optical modes, reducing the effective mode area, increasing the optical power density, and offering a wide evanescent optical field, which strengthens the interaction between photons and phonons. Beugnot et al. demonstrated the first complete measurement and numerical modeling of Brillouin scattering in a subwavelength-diameter optical fiber in 2014, as depicted in Figure 3a [58]. Contrary to standard single-mode fibers, the small waveguide boundary conditions enable micro/nanofibers to support new acoustic waveforms, including surface acoustic waves (SAWs) and hybrid acoustic waves (HAWs). SAWs propagate at a specific speed of 3,400 m s^−1^ on the surface of micro/nanofibers and generate new useful optical sidebands in the scattered spectrum with a frequency of approximately 6 GHz. In a further study, the authors investigated an optoacoustic spin–orbit interaction using Brillouin backscattering in a silica nanofiber [59] and reported the first measurement of SBS in silica nanofibers from both hybrid and surface acoustic waves [60]. In 2016, the perfect cancellation of Brillouin scattering arising from Rayleigh acoustic waves by engineering a silica nanofiber with exactly opposing photo-elastic and moving-boundary effects is realized by Florez et al., as shown in Figure 3b [61]. This work supports not only the case when the photo-elastic and moving-boundary effects act in opposite direction (resulting in cancellation) but also when the photo-elastic and moving-boundary effects act in the same direction (resulting in enhanced interaction). This is highly significant for the design of micro/nanophotonic devices based on photon–phonon interactions. Recently, the stimulated single-sideband Brillouin interactions with a strong coupling strength of approximately 300 W^−1^ m^−1^ and an ultranarrow linewidth response near 100 kHz in a homogeneous few-mode tapered fiber are reported (Figure 3c) [62].

The SBS effect in micro/nanofibers was further utilized for various applications, such as fiber taper diameter characterization, gas pressure sensing, temperature sensing, refractive index sensing, force sensing, and all-optical modulation [63], [64], [65], [66], [67], [68], [69]. From Ref. [58], it can be known that the acoustic frequencies in micro/nanofibers are closely associated with the fiber diameter. Thus, the diameter of a micro/nanofiber can be measured by the Brillouin frequency shift. In 2017, Godet et al. proposed to exploit the full elastic properties of tapered optical fibers and demonstrated a simple and accurate measurement technique of their diameters and their uniformity [63]. As shown in Figure 3d, the authors employed single-ended heterodyne coherent detection in combination with the backward Brillouin scattering to achieve a sensitivity as high as a few nanometers for fiber taper diameters ranging from 500 nm to 1.2 μm. Later, Jarschel et al. described an alternative method to characterize the micro/nanofiber diameter based on forward Brillouin scattering, with a resolution higher than 0.5 % [65]. Due to the high optical power density and wide evanescent optical field, micro/nanofibers are also appropriate for sensing applications. Gas pressure sensing, temperature sensing, refractive index sensing, and force sensing (Figure 3e) based on SBS in micro/nanofibers have been successively demonstrated [64], [66], [67], [68]. The combination of micro/nanofibers and novel materials is also an interesting research direction. Zhu et al. investigated all-optical modulation based on SBS in a graphene microfiber [69]. The authors found that the power of signal light decreases and the resonance wavelength shifts toward the blue with the increase of the pump power.

### 3.2 SBS in silica WGM microcavities

Silica WGM microcavities have drawn intense attention over the past decades. Due to the ultrahigh *Q* factor and small mode volume, silica WGM microcavities significantly enhance the light–matter interactions and reduce the threshold of nonlinear optical effects [70], [71]. SBS is an inelastic scattering process, which is induced by the coherent interaction between light photons and acoustic phonons. Therefore, the pump light, scattering light, and acoustic wave are all required to satisfy the resonance conditions of the microcavity. As the linewidth of Brillouin gain ranges from MHz to tens of MHz, fabricating a microcavity that fulfill these conditions poses a major challenge.

**Figure 4:**
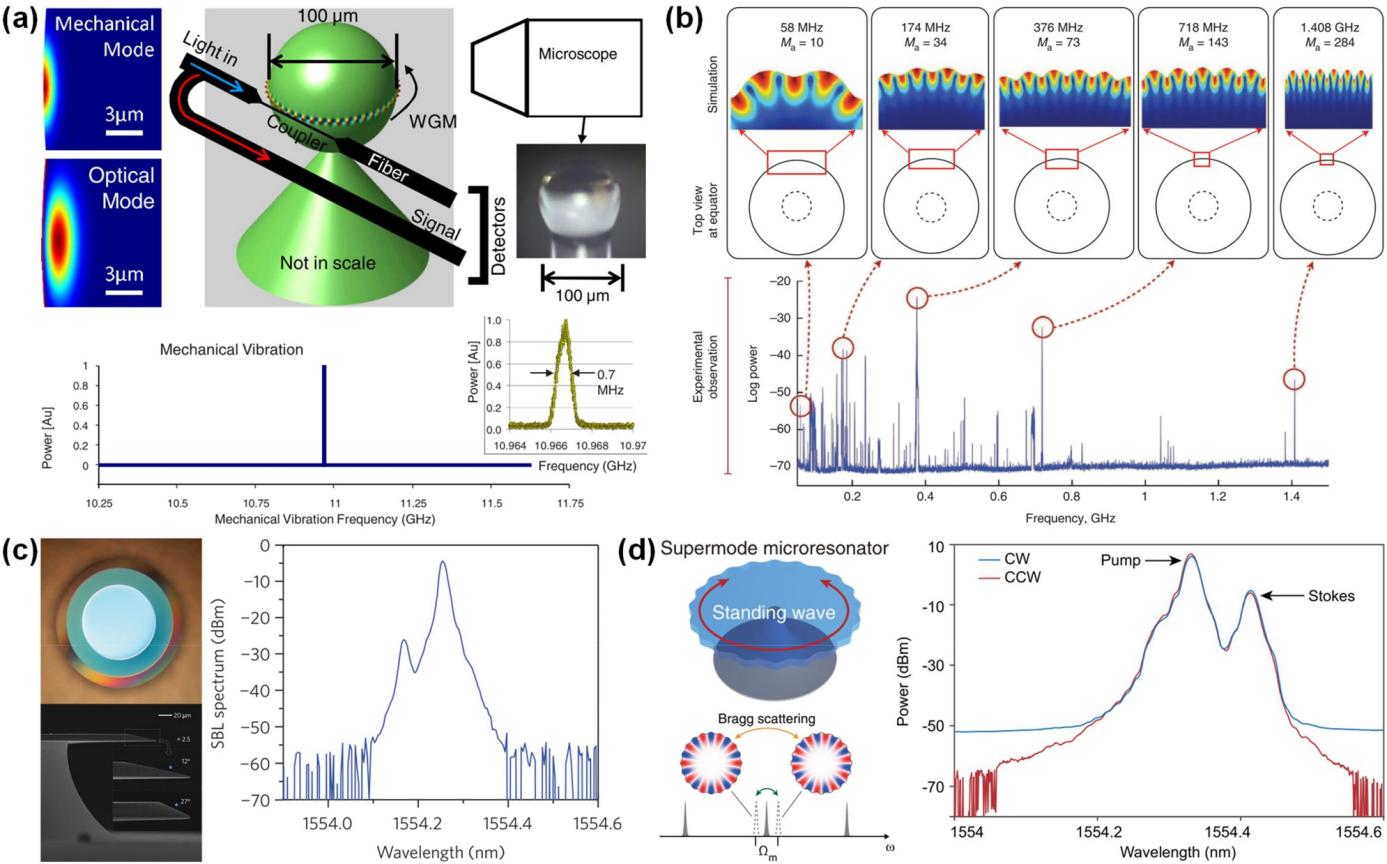
SBS in silica WGM microcavities. (a) Backward Brillouin scattering in a silica microsphere cavity. Reproduced with permission [53]. Copyright 2009, American Physical Society. (b) Forward Brillouin scattering in a silica microsphere cavity. Reproduced with permission [48]. Copyright 2011, Springer Nature. (c) On-chip SBS in a 1-mm-diameter silica wedge disk cavity. Reproduced with permission [22]. Copyright 2012, Springer Nature. (d) SBS in a silica supermode microcavity [73].

In WGM microcavities, there exist three potential solutions to this problem: (i) the resonance frequency spacing between two WGMs that pertain to different mode families is commensurate with the Brillouin frequency shift, e.g., highly multimode microcavity [48], [53]; (ii) the Brillouin frequency shift is an integral multiple of the FSR of the microcavity, e.g., FSR-matched microcavity [22]; or (iii) the resonance frequency spacing between a pair of supermodes is equal to the Brillouin frequency shift, e.g., supermode microcavity [72], [73]. In 2009, Tomes and Carmon observed backward Brillouin scattering for the first time in a highly multimode silica microsphere cavity by exploiting higher-order optical modes, as shown in Figure 4a [53]. Later, the authors reported the experimental excitation of mechanical resonances by using forward Brillouin scattering (Figure 4b) [48]. SBS was demonstrated on a chip for the first time in a FSR-matched silica wedge disk cavity with *Q* factor of 8.75 × 10^8^ in 2012 (Figure 4c) [22]. This platform also provided full compatibility of this important device class with conventional semiconductor processing. SBS was also achieved based on FSR matching in microrod and microtoroid cavities [74], [75]. In 2018, Honda et al. achieved 11-GHz mode splitting of supermodes in coupled silica microtoroid cavities by altering the gap distance between the two cavities, and subsequently demonstrated SBS [72]. Recently, Wang et al. employed Bragg scattering to fabricate a silica micron-sized supermode microdisk cavity, which induces very large Brillouin optomechanical coupling rates, and realized SBS in the same spatial modes, as depicted in Figure 4d [73]. Since the initial demonstration of SBS in a microcavity, numerous applications have been presented, including lasing [53], [72], [75], [76], [77], [78], [79], [80], [81], sensing [82], [83], [84], [85], [86], [87], [88], microcomb [79], [89], [90], [91], [92], microwave generation [46], [90], [91], induced transparency [50], [93], parity-time symmetry [94], [95], [96], isolator [95], [97], and light storage [50], [98].

**Figure 5:**
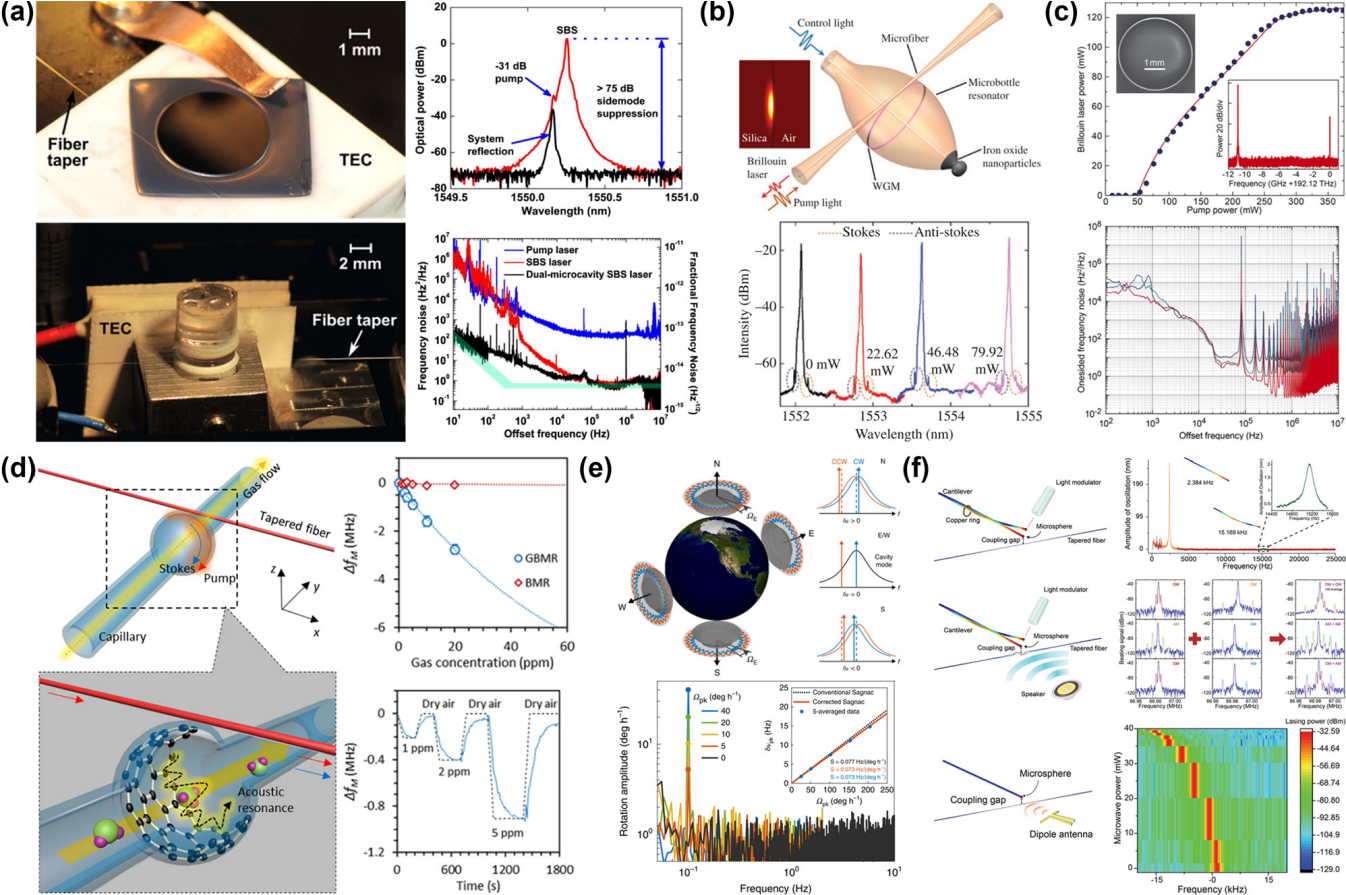
Lasing and sensing applications based on SBS in silica microcavities. (a) Narrow-linewidth Brillouin laser based on a microdisk cavity and a microrod cavity [78]. Copyright 2015, Optica Publishing Group. (b) Tunable Brillouin laser based on a hybrid microbottle cavity [81]. (c) High-power and low-noise Brillouin laser based on a microtoroid cavity [75]. Copyright 2022, Optica Publishing Group. (d) Highly sensitive detection of ammonia gas via a graphene inner deposited microbubble cavity [84]. (e) A monolithic Brillouin laser gyroscope via a microdisk cavity. Reproduced with permission [87]. Copyright 2020, Springer Nature. (f) Multiphysical sensing of light, sound, and microwave via a microsphere cavity [88].

The SBS possesses high gain and narrow gain bandwidth characteristics, rendering it an ideal platform for attaining high-performance lasers. Table 2 provides key parameters of stimulated Brillouin lasers (SBLs) on various photonic waveguide material platforms. In Ref. [53], the authors achieved a Brillouin laser with a threshold of 26 μW based a silica microsphere cavity. In 2012, Li et al. presented a low-threshold and ultralow-noise SBL at 1,550 nm from an ultrahigh-*Q* silica wedge disk cavity [76]. Shortly thereafter, the authors obtained a high-performance SBL at 1,064 nm [77]. As shown in Figure 5a, Loh et al. proposed a dual-microcavity Brillouin laser that leveraged one silica microdisk cavity to generate tunable 1,550 nm laser light via SBS and a second microrod cavity for frequency stabilization of the SBS light [78]. This configuration reduced the fractional frequency noise to 7.8 × 10^−14^ Hz^−1/2^. SBL was also demonstrated in microbottle, microbubble, and microtoroid cavities [75], [79], [80], [81]. Through an ultrahigh-*Q* hybrid microbottle cavity, Zhu et al. realized a tunable SBL with a wavelength tuning range of 2.68 nm (Figure 5b) [81]. Recently, a high-power and low-noise SBL based on a single microtoroid cavity was achieved with a maximum output power of 126 mW and a fundamental linewidth of 245 mHz (Figure 5c) [75]. Besides, the researchers had carried out a series of studies on the linewidth, thermal characteristics, and frequency stability of Brillouin lasers [99], [100], [101], [102].

**Table 2:** Key parameters of SBLs on various photonic waveguide material platforms.

Ref.	Material platform	Resonator structure	Operation wavelength	Brillouin frequency shift	Lasing threshold	Output power	Laser linewidth
[54]	CaF_2_	Microrod	1,064 nm	17.5 GHz	3 μW	–	–
[53]	SiO_2_	Microsphere	1,550 nm	11 GHz	26 μW	–	–
[103]	As_2_S_3_	Fabry–Pérot cavity	1,550 nm	7.67 GHz	500 mW	–	–
[76]	SiO_2_	Microdisk	1,550 nm	11 GHz	40 μW	1.4 mW	0.2 Hz
[104]	As_2_S_3_	Fiber ring	1,550 nm	7.5 GHz	360 mW	15 mW	100 kHz
[77]	SiO_2_	Microdisk	1,064 nm	15.9 GHz	1 mW	1.8 mW	0.3 Hz
[105]	BaF_2_	Microdisk	1,550 nm	8.27 GHz	7.1 mW	1.3 mW	27 kHz
[78]	SiO_2_	Microdisk	1,550 nm	11 GHz	100 μW	15 mW	2 Hz
[79]	SiO_2_	Microbottle	1,550 nm	11 GHz	450 μW	–	–
[80]	SiO_2_	Microbubble	1,550 nm	11 GHz	420 μW	0.3 μW	–
[72]	SiO_2_	Coupled microtoroid	1,550 nm	11 GHz	50 mW	100 μW	–
[51]	Si	Microring	1,550 nm	6.03 GHz	10 mW	170 μW	20 kHz
[81]	SiO_2_	Microbubble	1,550 nm	11 GHz	170 μW	1 μW	–
[106]	As_2_S_3_	Microring	1,550 nm	7.74 GHz	530 μW	180 μW	–
[107]	Si_3_N_4_	Microring	674 nm	25.11 GHz	14.7 mW	9.28 mW	269 Hz
[75]	SiO_2_	Microtoroid	1,550 nm	11 GHz	49 mW	126 mW	0.245 Hz
[108]	Si_3_N_4_	Microring	1,550 nm	10.8 GHz	380 µW	80 μW	–
[109]	MgF_2_	Microdisk	1,550 nm	13.47 GHz	1.8 mW	–	–
[110]	Si_3_N_4_	Microring	1,550 nm	10.8 GHz	2.3 mW	11 mW	0.1 Hz
[111]	GeSbS	Microring	1,550 nm	7.49 GHz	24.8 mW	1.56 mW	8 kHz
[112]	GeSbS	Microring	1,550 nm	7.5 GHz	960 μW	0.4 mW	58 Hz
[113]	Si_3_N_4_ and TeO_2_	Microring	1,550 nm	8.1 GHz	47 mW	2 mW	7 Hz
[26]	LiNbO_3_	Microring	1,550 nm	8.57 GHz	100 mW	5.6 mW	9.4 Hz

As the SBS frequency shift is highly sensitive to environmental variables and the Brillouin laser possesses narrow linewidth and low noise, SBL has great advantages in sensing applications. In 2013, Bahl et al. had demonstrated that the liquid sensing, which was achieved via forward Brillouin scattering in a microbubble cavity, held great potential for application [82]. Based on this research, the authors accomplished a rapid photonic sensing of the mechanical properties of freely flowing particles in a fluid [83]. Forward Brillouin scattering in microbubble cavities can also be employed for gas sensing. Yao et al. reported a highly sensitive detection of ammonia gas with a noise equivalent detection limit of 1 ppb via a graphene inner deposited microbubble cavity, as shown in Figure 5d [84]. Recently, the authors realized a microlaser sensor for high sensitivity multispecies gas detection, by exciting multiple Brillouin lasers in a graphene-functionalized silica microsphere cavity [85]. In 2017, Li et al. verified a chip-based optical gyroscope that uses counterpropagating Brillouin lasers to measure rotation as a Sagnac-induced frequency shift [86]. Later, the authors reported a monolithic Brillouin laser gyroscope, which can measure sinusoidal rotations with amplitudes as small as 5 deg h^−1^ and Earth’s rotation. The device had an angle random walk noise of 0.068 deg h^−1^ and bias instability of 3.6 deg h^−1^ (Figure 5e) [87] As depicted in Figure 5f, multiphysical sensing of light, sound, and microwave was also explored by using the Brillouin lasing in a silica microsphere cavity [88].

**Figure 6:**
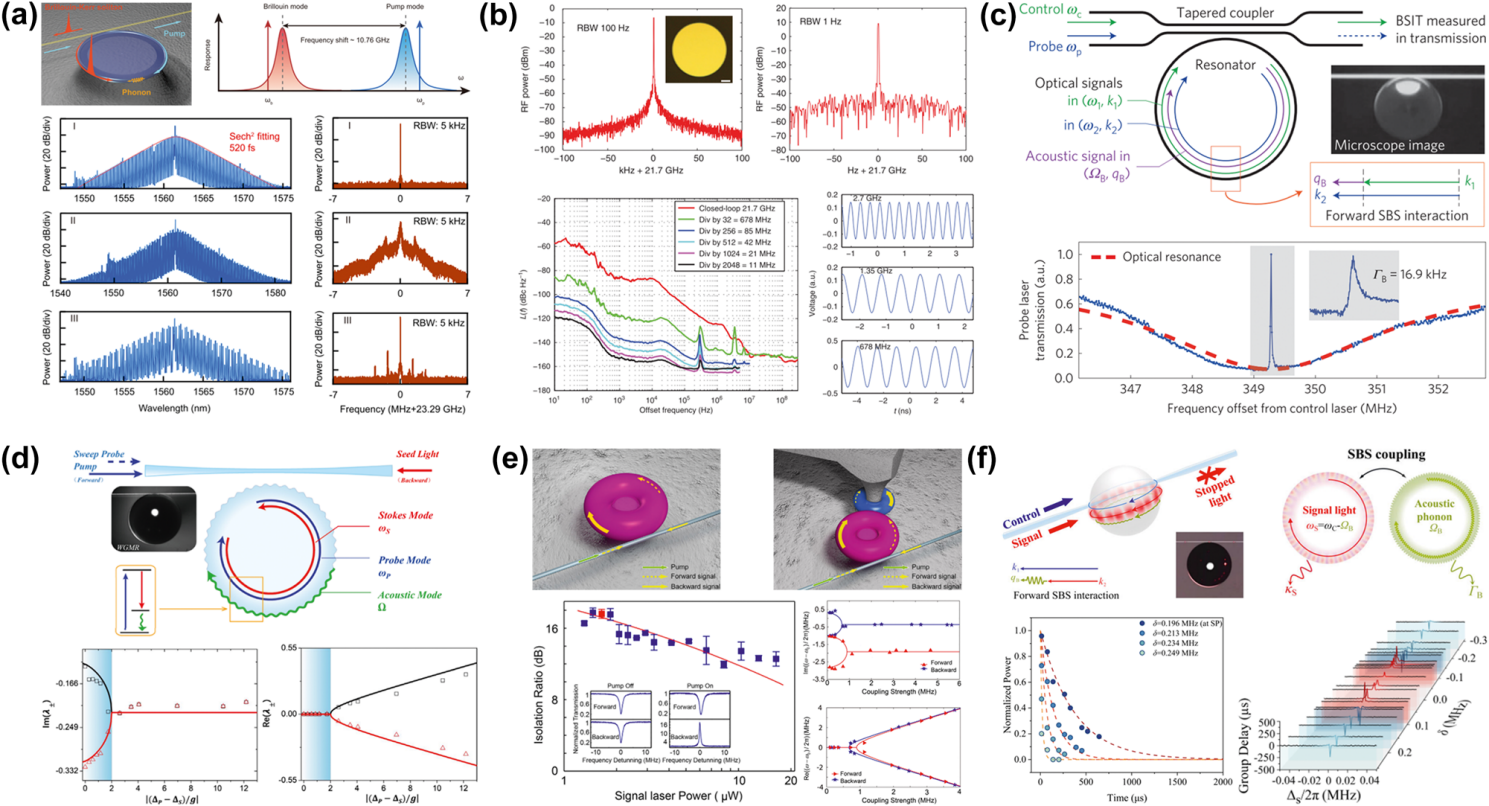
Optical microcomb, microwave generation, induced transparency, parity-time symmetry, optical isolator, and light storage applications based on SBS in silica microcavities. (a) Brillouin–Kerr soliton and microwave signal source based on a microtoroid cavity [91]. (b) Frequency synthesis by the K-band low-noise Brillouin microwave oscillator [46]. (c) Brillouin scattering induced transparency based on a microsphere cavity. Reproduced with permission [93]. Copyright 2015, Springer Nature. (d) Synthetic anti-PT symmetry in a single microcavity. Reproduced with permission [94]. Copyright 2020, American Physical Society. (e) Optical isolator and nonreciprocal parity-time symmetry based on a microtoroid cavity. Reproduced with permission [95]. Copyright 2020, Wiley-VCH Verlag. (f) Optical storage at storing light point based on a microsphere cavity. Reproduced with permission [98]. Copyright 2024, Springer Nature.

Due to the high *Q* factor of WGM microcavities and the high gain of SBS, it is feasible for the interaction of the Kerr microcomb with SBS. In 2006, Asano et al. observed Brillouin-coupled four-wave-mixing in an ultrahigh-*Q* silica microbottle cavity [79]. Similar phenomena had been observed in a microbubble cavity [89]. Recently, Bai et al. reported a Brillouin-Kerr soliton microcomb with narrow-linewidth comb lines and stable repetition rate based on a microdisk cavity [90]. Later, the authors realized a strong interaction between the generated soliton comb and the background light in a Brillouin–Kerr microcomb system based on a microtoroid cavity (Figure 6a) [91]. Besides, Zhang et al. demonstrated spatial multiplexing of soliton microcombs by combining Kerr and Brillouin nonlinearities in a silica microsphere cavity [92]. This demonstration provided an ideal scheme for realizing highly coherent dual-comb sources in a compact, low-cost, and energy-efficient manner, with uniquely low beating noise.

In addition to the aforementioned, SBS in silica microcavities has several other applications. In 2013, Li et al. reported generation of highly coherent microwaves using a compact, on-chip Brillouin laser in a microdisk cavity (Figure 6b) [46]. Recently, researchers had realized turnkey ultralow-noise microwave signal source in a microtoroid cavity (Figure 6a) [91]. In 2015, Kim et al. demonstrated a Brillouin scattering induced transparency phenomenon generated by acousto-optic interaction of light with long-lived propagating phonons in a silica microsphere cavity (Figure 6c) [93]. In the same year, Dong et al. also observed the phenomenon and demonstrated nonreciprocal optical storage in a microsphere cavity [50]. In 2020, Zhang et al. demonstrated synthetic anti-PT symmetry in a spectral dimension induced by nonlinear Brillouin scattering in a single microcavity (Figure 6d) [94]. As shown in Figure 6e, Ma et al. reported a chip-based, tunable all-optical isolator based on SBS in a microtoroid cavity and accomplished the nonreciprocal parity-time symmetry induced by SBS in two directly coupled microtoroid cavities [95]. Based on an anomalous gauge potential, where near-phase-matched nonlinear Brillouin scatterings enable such unique direction-dependent gauge phases, Yang et al. construct photonic isolators in the frequency domain [97]. Later, Chen et al. demonstrated a parity-time transition with an unusual memory effect near an exceptional point in an SBS laser system (Figure 6e) [96]. The authors also achieved a room-temperature storing light scheme in a chip-scale 90-μm-radius microcavity (Figure 6f) [98].

## 4 Chalcogenide glasses

The increasing sophistication of integrated photonics technology makes it possible to construct photonic circuits on the chip surface and to modulate nonlinear effects, among other functionalities [114], [115], [116], [117], [118]. Even though SBS has been extensively studied in micro/nanofibers and microresonators, recently there has been a growing interest among researchers to utilize SBS in integrated photonic circuits [27], [107], [110], [119], [120], [121], [122]. The ability to control the interactions between photons and phonons in chip-sized devices (rather than in tens of meters of optical fibers) promises not only to lead to new physical phenomena at the micro- and nanoscale but also to open the way for the realization of key on-chip technologies. Silica has emerged as the preferred material for technologies such as SBS-based distributed temperature and strain sensors, owing to its ease of stretchability and cost-effectiveness [123]. However, silica-based platforms often exhibit larger device sizes and lower integration levels due to the smaller differences in waveguide refractive indices. As a result, attention has turned to chalcogenide glasses (ChGs) for the realization of on-chip SBS, leveraging their exceptional Brillouin gain coefficients [124].

The simultaneous confinement of light and acoustic waves in the integrated platform is the first prerequisite for the realization of Brillouin applications [125]. Chalcogenide-based glass materials are composite compounds consisting of one or more elements in the VIA group (mainly including sulfur (S), selenium (Se), and tellurium (Te)). These materials exhibit an ultrawide infrared transparency window ranging from 0.5 to 25 μm [126], high linear refractive indices between 2 and 3.5 [127], [128], and nonlinear refractive indices of (2–20) × 10^−18^ m^2^/W [129], [130]. Additionally, they have a low elastic modulus of less than 20 GPa [131]. This combination of properties allows for the confinement of both optical and elastic total internal reflection waves. In recent years, As_2_S_3_ and GeSbS have emerged as the most widely utilized chalcogenide glass materials for achieving SBS in integrated platforms. This article will provide an in-depth introduction to the SBS effect and its various applications based on these two materials.

### 4.1 SBS in integrated As_2_S_3_ waveguides and resonators

**Figure 7:**
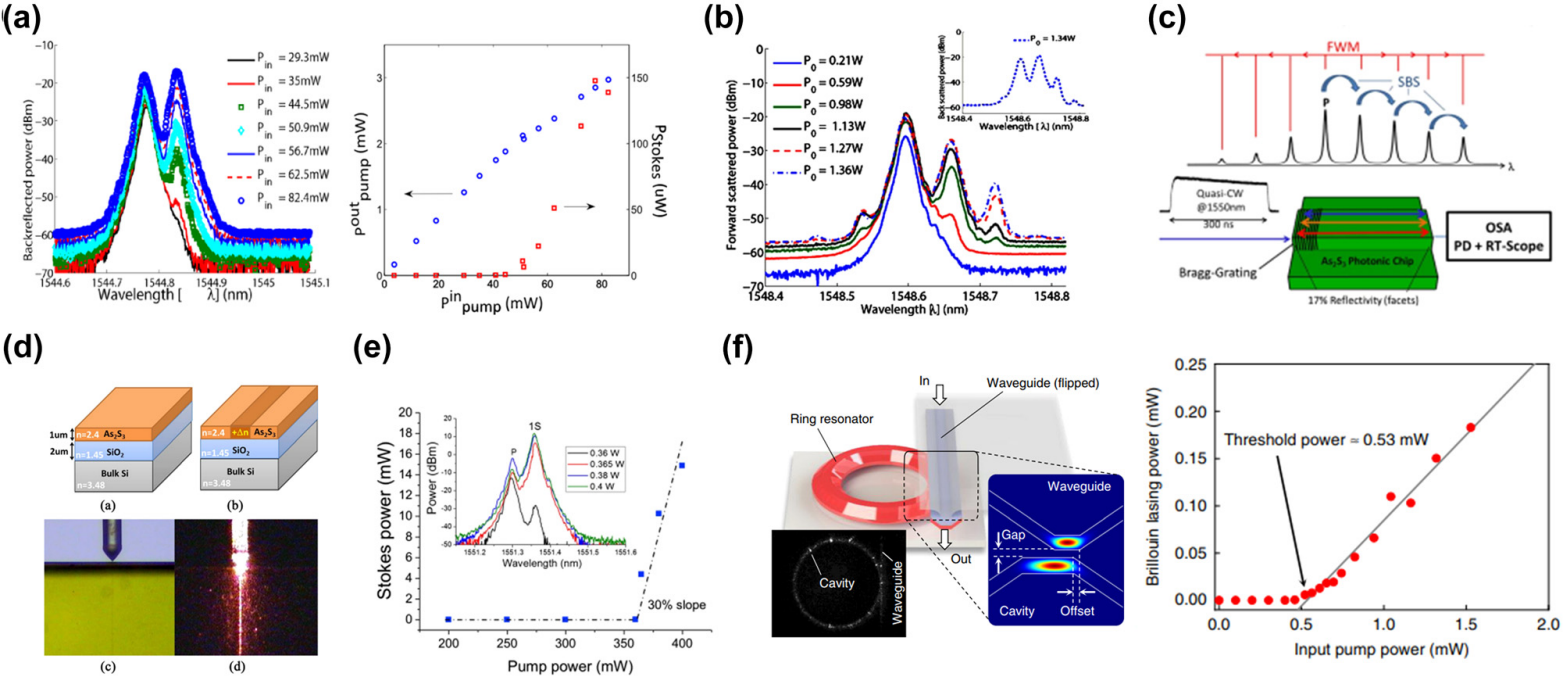
SBS in As_2_S_3_ waveguides and resonators. (a) Characterization of on-chip SBS [24]. Copyright 2011, Optica Publishing Group. (b) Multiorder Stokes generation via the cascaded SBS process in an optical chip [103]. Copyright 2011, Optica Publishing Group. (c) Phase-locked optical frequency comb generation [132]. Copyright 2014, Optica Publishing Group. (d) Directly written As_2_S_3_ waveguides [133]. Copyright 2012, Optica Publishing Group. (e) Narrow linewidth Brillouin laser based on As_2_S_3_ waveguides and fiber ring cavity [104]. Copyright 2013, Optica Publishing Group. (f) Trapezoidal As_2_S_3_ waveguide structure and input power versus Brillouin lasing power [106].

In 2011, Pant et al. demonstrated SBS in a 7-cm long As_2_S_3_ chalcogenide rib waveguide (Figure 7a), which was the first instance of on-chip SBS realization. [24]. The measured Brillouin frequency shift and linewidth were 7.7 GHz and 34 MHz, respectively. Additionally, the probe gain of 16 dB was obtained at a CW pump power of ∼300 mW. In further research, Pant et al. discovered that reflections from the front and back end faces of the chip created a Fabry–Perot cavity, which reduced the SBS gain threshold, enabling multi-Stokes generation at reduced pump powers [103]. When a laser with a pulse width of 2 μs and a peak power of 1.34 W was utilized as the pump source, third-order Stokes spectral lines were successfully observed (Figure 7b). Furthermore, Büttner et al. reported the generation of phase-locked, 7.5 GHz-repetition-rate optical frequency combs in an on-chip Fabry–Perot waveguide resonator incorporating a Bragg grating (Figure 7c) [132]. Recently, Choudhary et al. achieved remarkable on-chip SBS gain of 52 dB in centimeter-scale As_2_S_3_ rib waveguides, demonstrating that performance comparable to kilometers of optical fiber can be realized in integrated photonic devices [124]. In previous study, As_2_S_3_ waveguides were fabricated using reactive ion etching (RIE). In contrast, Levy et al. utilized laser direct writing to produce centimeter-long As_2_S_3_ waveguides (Figure 7d) and similarly observed SBS amplification of probe waves [133].

Previous research has demonstrated that integrated As_2_S_3_ waveguides can provide significant Brillouin gain, enabling the transfer of nearly all SBS applications previously conducted on spatial platforms to integrated platforms. Currently, SBS in integrated As_2_S_3_ waveguides has been applied in various fields, including narrow-linewidth laser, optical communication, optical signal processing, and microwave photonics. In the following sections, this article will highlight some representative works in this area.

By using a 7-cm As_2_S_3_ waveguide as the laser gain medium and combining it with a fiber ring cavity, Kabakova et al. achieved a narrow-linewidth, waveguide-based Brillouin laser for the first time [104]. The lasing threshold and the slope efficiency were 360 mW and 30 %, respectively (Figure 7e). Although the Brillouin laser mentioned above used an integrated As_2_S_3_ waveguide as the gain medium, it still requires a fiber ring cavity for feedback. Therefore, it is not a fully on-chip integrated Brillouin laser. In 2017, Morrison et al. developed a compact spiral device within a silicon circuit, achieving a tenfold improvement in Brillouin amplification [134]. Subsequently, they created a ring resonator with a FSR precisely aligned with the Brillouin frequency shift, leading to the first demonstration of Brillouin lasing in a planar integrated circuit. However, the Brillouin lasing threshold in this work was relatively high, at approximately 50 mW. To address this issue, Kim et al. proposed and fabricated a trapezoidal As_2_S_3_ waveguide (Figure 7f) [106]. The design of trapezoidal waveguide significantly reduced the propagation loss of the waveguide, successfully increasing the *Q* factor of the microring resonator to 10^7^. This improvement allowed the threshold power for SBS lasing to be as low as 0.54 mW, which is 100 times lower than previous records.

**Figure 8:**
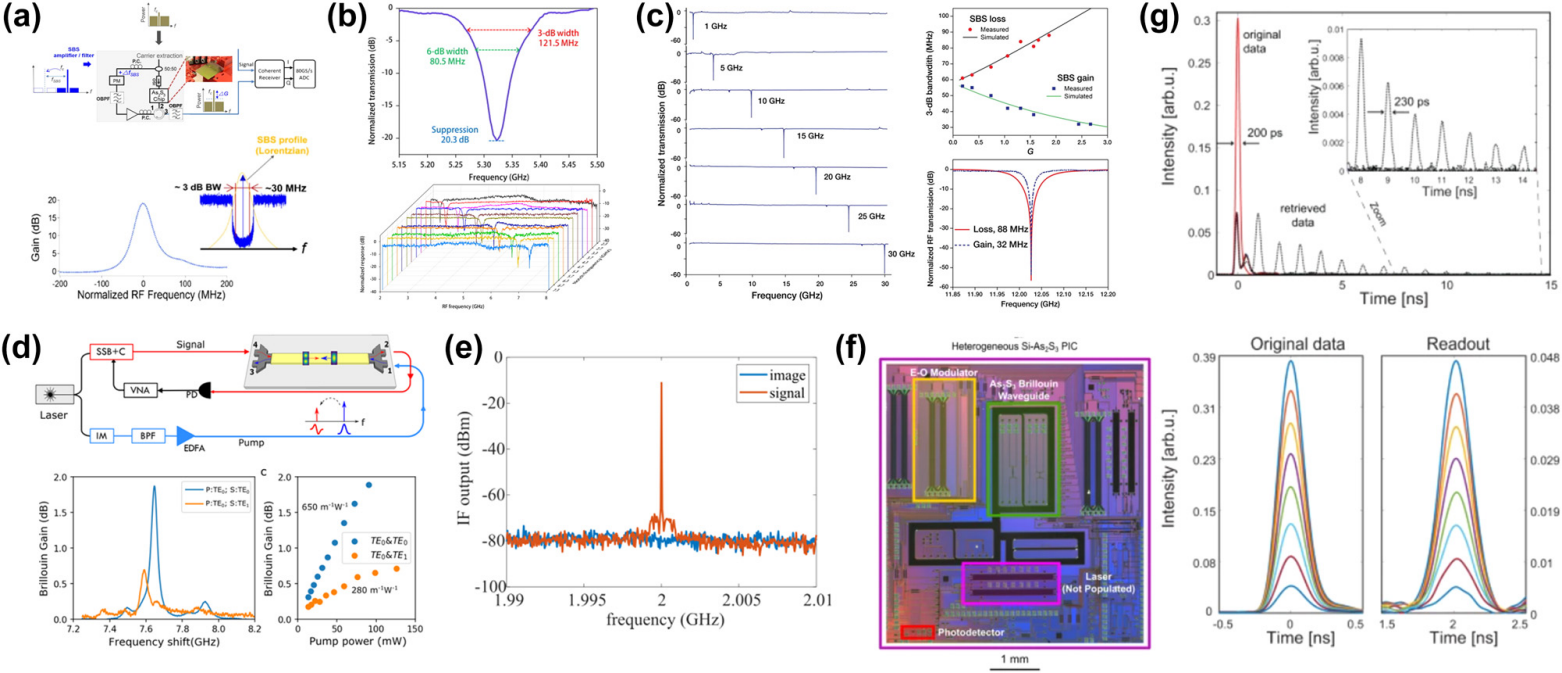
SBS photonic devices based on As_2_S_3_ waveguides. (a) On-chip SBS-based self-coherent optical orthogonal frequency-division multiplexing (self-CO-OFDM) [135]. (b) Tunable MWPNF based on SBS in a As_2_S_3_ photonic chip [136]. (c) Low-power, chip-based SBS microwave photonic filter [137]. Copyright 2015, Optica Publishing Group. (d) Experimental demonstration of the circulator-free Brillouin photonic circuit [138]. (e) Chip-based MWP mixer with image rejection of broadband signals [139]. Copyright 2023, Optica Publishing Group. (f) Heterogeneous Si–As_2_S_3_ photonic integrated circuit [140]. (g) Tunable storage of a 200 ps-long data pulse for up to 14 ns [146].

Modern coherent optical communication employs advanced spectrally efficient modulation formats, which require complex narrow-linewidth local oscillators (LOs). To address this issue, in 2018, Giacoumidis et al. proposed to harness large-gain SBS on an integrated As_2_S_3_ photonic chip for ultrahigh-resolution selective filtering for carrier recovery in high-capacity self-coherent optical signals, eliminating the need for separate LOs [135]. Due to the narrow linewidth of SBS and the “self-referencing” feature of the proposed technique, a record-breaking narrow carrier guard band of ∼265 MHz for a data rate of 116.82 Gbit s^−1^ is achieved for self-coherent optical signals (Figure 8a). Additionally, SBS offers a pathway to achieving megahertz-resolution integrated microwave photonics (MWP) filters, which can exhibit line widths of 10–100 MHz, a resolution unmatched by most on-chip devices. Morrison et al. reported the first demonstration of a tunable microwave photonic notch filter (MWPNF) based on SBS in a As_2_S_3_ photonic chip [136]. The photonic chip-based MWPNF with a 3-dB bandwidth of 12 MHz, a notch depth of 20 dB, and frequency tunability in the range of 2–8 GHz (Figure 8b). While the feasibility of on-chip SBS filters has been demonstrated, the need for substantial SBS gain and significant pump power to achieve the desired filter suppression poses a significant challenge to their development. Marpaung et al. improved the SBS MWP bandstop filter scheme in centimeter-scale As_2_S_3_ waveguides [137]. By leveraging ultralow Brillouin gain (1–4 dB) from a compact photonic chip and a novel approach of optical resonance-assisted RF signal cancellation, they achieved the first chip-based MWP bandstop filter with ultra-high suppression (>50 dB), high resolution (32–88 MHz) in the megahertz range, and 0–30 GHz frequency tuning (Figure 8c). Achieving fully integrated Brillouin photonic circuits necessitates the separation of pump and signal waves following Brillouin interactions, which is crucial to prevent crosstalk and to safeguard critical components like lasers and photodetectors. As a result, for backward SBS devices, nonreciprocal elements such as circulators are conventionally required, which poses challenges and increases complexity. To address this issue, Liu et al. utilized backward intermodal Brillouin scattering to achieve an As_2_S_3_–Si hybrid multimodal photonic circuit (Figure 8d), with a Brillouin gain coefficient of 280 m^−1^ W^−1^ (two orders of magnitude improvement over conventional optical fibers) [138]. Frequency mixers play a vital role in radio frequency (RF) front-ends by downconverting RF signals to lower frequencies. McKay et al. demonstrated the first chip-based MWP mixer with image rejection of broadband signals utilizing SBS and interferometry [139]. They successfully demonstrated frequency down-conversion for carrier frequencies between 10 GHz and 16 GHz, along with ultrahigh image rejection of up to 70 dB for a single tone (Figure 8e). In previous work, chalcogenide waveguides can only be used as a standalone element in MWP systems, while key MWP components like electro-optic (E-O) modulators and photodetectors (PDs) remain off-chip. However, to reduce the size, weight, and power of Brillouin-based MWP processors, it is crucial to integrate chalcogenide Brillouin circuits with the necessary components of an MWP link, including E-O modulators and PDs. Garrett et al. created a compact MWP processing platform (Figure 8f) with high spectral resolution by heterogeneously integrating As_2_S_3_ Brillouin waveguides into a silicon photonic platform, which includes active E-O modulators and PDs [140]. Furthermore, they demonstrated an integrated MWP notch filter using on-chip silicon devices and Brillouin gain in As_2_S_3_ waveguides. This filter achieved an out-of-band rejection of 51 dB, a 3-dB bandwidth of 37 MHz, and tunable notch central frequency spanning over 15 GHz. Another important application of SBS is the storage or delay of optical signals. Currently, Brillouin-based memory has been implemented in highly nonlinear fibers [141] as well as integrated photonic circuits [142], [143], [144], [145]. Limited by the phonon lifetime, Brillouin-based memory is generally applicable to continuous-wave or pulsed optical signals longer than a few nanoseconds. Recently, Stiller et al. experimentally demonstrated Brillouin interactions at the 150-ps time scale and a delay for a record 15 ns, which corresponds to a delay of 100 pulse widths, which was enabled by the high local gain of the chalcogenide waveguides as the optoacoustic interaction length reduces with the pulse width (Figure 8g) [146].

### 4.2 SBS in integrated GeSbS waveguides and resonators

**Figure 9:**
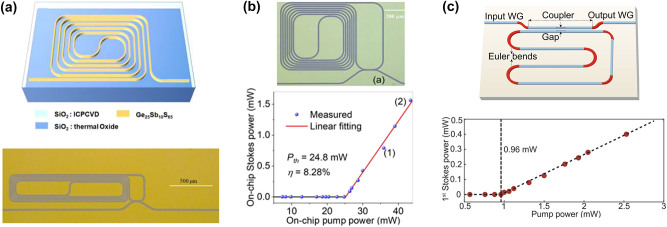
SBS in GeSbS waveguides and resonators. (a) A 7 cm-long compact spiral GeSbS waveguide and a high-*Q* microring resonator [150]. (b) Brillouin laser power versus on-chip pump power [111]. (c) A finger-shaped GeSbS microresonator with Euler bends and the ultralow-threshold Brillouin laser [112]. Copyright 2024, Optica Publishing Group.

While integrated As_2_S_3_ waveguides have exhibited superior SBS performance and have been extensively used, the oxidation sensitivity of As leads to a reduced laser damage threshold, limiting their applications in lasers and other areas [147]. Furthermore, As is toxic and environmentally unfriendly. By substituting As with Sb and incorporating Ge, a ternary glass system can be developed, resulting in a nontoxic, arsenic-free ChG (Ge-Sb-As). This material has been proven to be well-suited for optical properties and glass thin films [148], [149]. In recent years, advancements in micro- and nanofabrication technologies have led to notable achievements in the generation of SBS using integrated GeSbS waveguides. In 2021, Song et al. conducted the first experimental characterization of SBS in low-loss integrated GeSbS waveguides [150]. In this work, they fabricated a 7 cm-long spiral waveguide with a propagation loss as low as 0.2 dB/cm, along with a microring resonator featuring a high loaded *Q* factor of 1.34 × 10^6^ (Figure 9a). The measured Brillouin linewidth was 47.8 MHz, and the gain coefficient was 338 m^−1^ W^−1^, comparable to that of As_2_S_3_ chalcogenide. Subsequently, Song et al. designed and fabricated a high-*Q* spiral-ring resonator with a FSR that closely aligns with the Brillouin frequency shift. This alignment facilitated the generation of Brillouin laser. The laser exhibited an output power of 1.56 mW, a linewidth of 8 kHz, and a threshold of 24.8 mW (Figure 9b) [111]. To further decrease the threshold for Brillouin lasers, Li et al. incorporated Euler bends, resulting in a finger-shaped GeSbS microresonator with a *Q* factor of 5.19 × 10^6^ (Figure 9c) [112]. The high Brillouin gain of GeSbS material, combined with the high *Q* factor of the microresonator, enables the generation of Brillouin lasers with a low threshold of 0.96 mW and a fundamental linewidth of 58 Hz.

## 5 Silicon

Silicon is one of the most promising platforms for building photonic integrated circuits (PIC) [151], [152], [153], [154], [155], which is compatible with modern CMOS nanofabrication facilities. Hence, investigating the SBS effect within silicon waveguides holds substantial importance. However, while conventional nanoscale silicon waveguides are adept at enhancing third-order Kerr and Raman nonlinearities, they struggle to generate efficient Brillouin nonlinear interactions [156]. Two primary factors contribute to the diminished SBS in silicon waveguides. Firstly, the photoelastic tensor component (*p*_12_) plays a crucial role in facilitating backward-SBS interactions by bridging transverse electromagnetic fields with longitudinal acoustic waves. However, in silicon, this component is notably diminished, being roughly 16 times less than what is found in silica [27]. Secondly, typical silicon-on-insulator (SOI) waveguides provide only weak acoustic confinement because there is little elastic mismatch between the silicon core and the silicon dioxide substrate. As a result, the generated sound fields typically radiate into the substrate instead of interacting with light, thereby limiting the occurrence of significant light-sound scattering [21], [42], [125]. Recently, it has been revealed that novel mechanisms for Brillouin coupling arise when light is confined below the optical wavelength scale. Such interactions result from the strong interaction of light with waveguide boundaries caused by radiation pressure. Recent experiments have shown that efficient Brillouin nonlinear interactions can be realized by harnessing radiation pressure, together with coupling to different photoelastic tensor components, in suspended or quasi-suspended silicon waveguide [21], [42]. Recently, with further advancements in research, SBS has been successfully demonstrated in both nonsuspended subwavelength antenna arrays [157] and ultralow-loss thick-SOI platforms [158]. This article will present a comprehensive analysis of Brillouin scattering phenomena within silicon-based nanoscale waveguides, particularly highlighting recent progress in amplification efficiency and demonstrating practical implementations across various domains.

### 5.1 Brillouin-active integrated silicon waveguides and resonators

**Figure 10:**
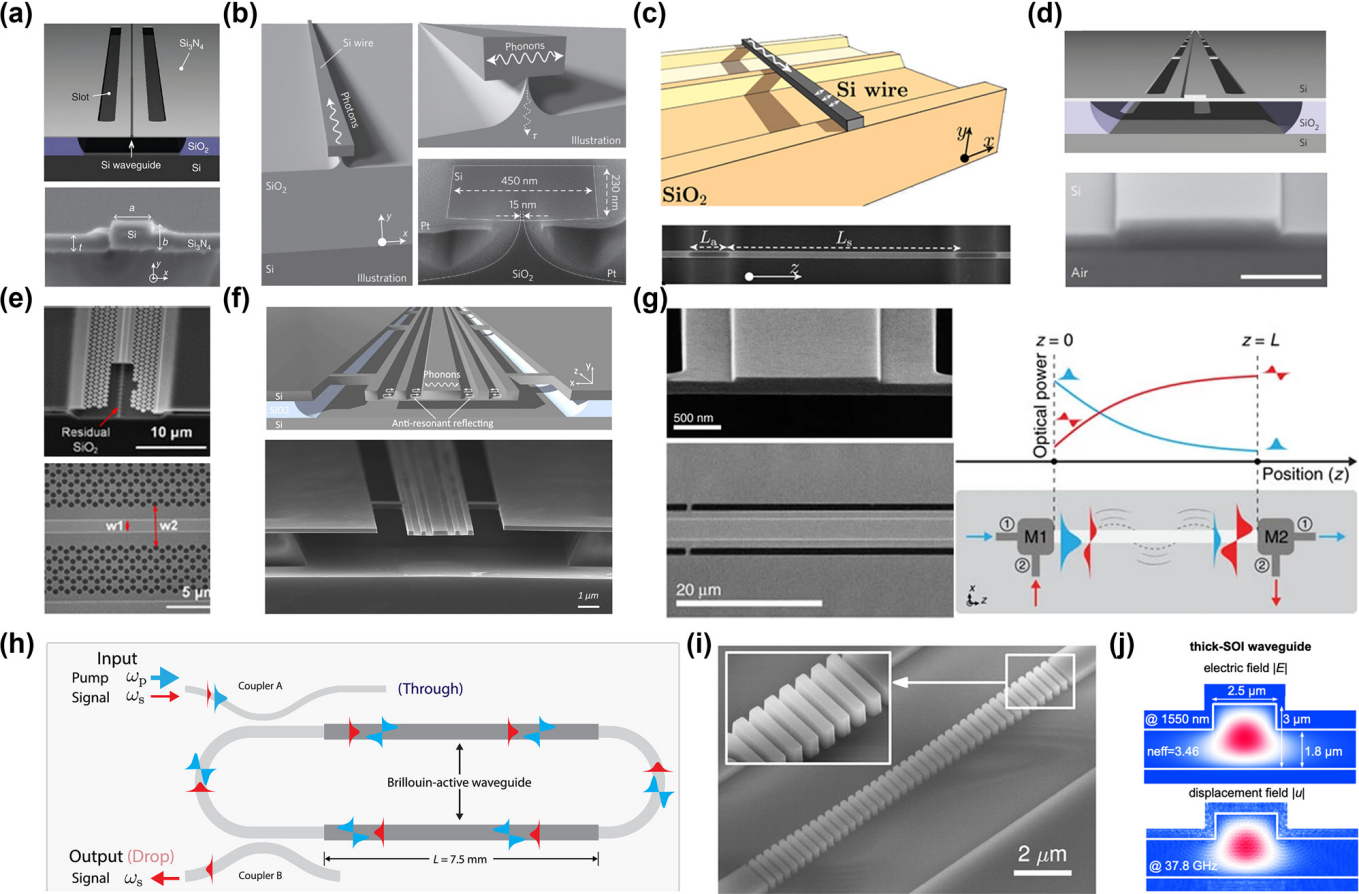
SBS in silicon waveguides and resonators. (a) Silicon waveguide on silicon nitride [37]. (b) Silicon pedestal with silica support. Reproduced with permission [23]. Copyright 2015, Springer Nature. (c) Suspended silicon waveguide [159]. (d) Membrane-suspended silicon waveguide. Reproduced with permission [160]. Copyright 2016, Springer Nature. (e) Hybrid photonic–phononic silicon waveguides [167]. (f) Suspended antiresonant acoustic waveguides [171]. (g) Multimode optomechanical waveguide [161]. (h) Suspended silicon racetrack resonator [162]. (i) Subwavelength silicon waveguide [157]. (j) Thick silicon waveguide [158].

**Figure 11:**
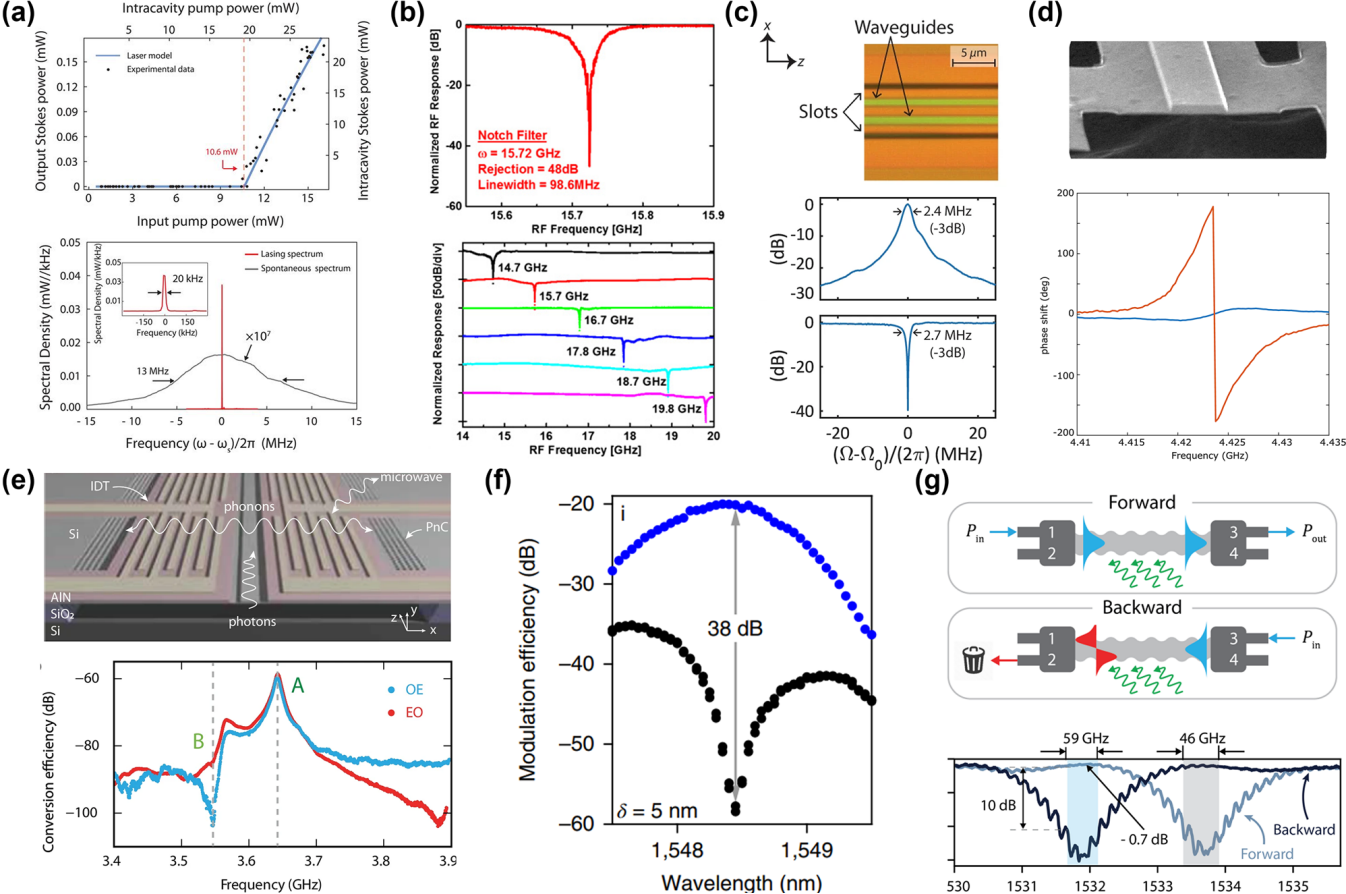
Applications enabled by silicon-based SBS (a) Brillouin laser. Reproduced with permission [51]. Copyright 2018, The American Association for the Advancement of Science. (b) MWP filter with a 48-dB suppression ratio, a 98-MHz bandwidth, and a 6-GHz frequency tuning range [173]. Copyright 2015, Optica Publishing Group. (c) MWP filter featuring a rejection ratio of 57 dB, a bandwidth of 2.7 MHz, and a frequency tuning range up to 6 GHz [174]. (d) 360° broadband Brillouin-based RF phase shifter [175]. (e) Microwave measurement. Reproduced with permission [176]. Copyright 2024, Springer Nature. (f) Nonreciprocal single-sideband modulation and mode conversion. Reproduced with permission [45]. Copyright 2018, Springer Nature. (e) SBS-based isolator [177].

To realize strong Brillouin nonlinear interactions within SOI waveguides, it is crucial to effectively confine the acoustic modes. Recently, a variety of innovative waveguide designs have been introduced, which primarily rely on three strategies. The first approach is to isolate the waveguide from the substrate, thereby effectively preventing acoustic leakage [23], [37], [159], [160], [161], [162], [163], [164], [165]. The second solution employs phononic bandgaps to impede the propagation of acoustic waves [166], [167], [168], [169]. The third strategy utilizes antiresonant acoustic waveguides, which can effectively confine phonons [170], [171], [172]. Then, this article will outline several groundbreaking and noteworthy contributions to the field. Shin et al. demonstrated SBS in silicon waveguides, for the first time, through a new class of hybrid photonic–phononic waveguides (Figure 10a) [37]. A total effective forward SBS gain coefficient of 2,750 W^−1^ m^−1^ was achieved, which is more than an order of magnitude higher than the gain coefficient obtained when considering the Brillouin nonlinearities of silicon as a bulk medium. To further enhance Brillouin gain, it is crucial to improve the confinement capability of silicon waveguides for acoustic waves. Researchers have made significant efforts in this area. Van Laer et al. demonstrated an exceptionally large overlap between near-infrared light and gigahertz sound waves that are colocalized in a quasi-suspended silicon waveguide (Figure 10b) [23]. This waveguide is propped up by a thin pedestal, effectively sealing off avenues for external phonon leakage and confining 10 GHz phonons within a footprint of less than 0.1 μm^2^. The structure not only boasts an impressive Brillouin gain coefficient of 3,218 W^−1^ m^−1^ but also supports a centimeter-scale Brillouin-active interaction length. Subsequently, Van Laer et al. attained a remarkable Brillouin gain coefficient of 6,561 W^−1^ m^−1^, surpassing the optical loss in a series of suspended silicon waveguides (Figure 10c) [159]. This achievement sets a solid foundation for the development of on-chip Brillouin optical amplifiers. Despite the strong Brillouin nonlinearity generated in the aforementioned new structures, achieving net gain remains a significant challenge due to nonlinear loss and free-carrier effects. Kittlaus et al. designed and fabricated a membrane-suspended silicon waveguide (Figure 10d) [160]. Due to the ultralow propagation losses (<0.2 dB cm^−1^) and strong Brillouin coupling (gain coefficient *G*_B_ > 10^3^ W^−1^ m^−1^) of this structure, large Brillouin amplification in silicon was realized for the first time. This resulted in amplification levels exceeding 5 dB with modest pump powers and demonstrated a record low threshold of 5 mW for net amplification. Wang et al. designed a class of hybrid photonic–phononic silicon waveguides (Figure 10e) [167], which combine the advantages of a suspended silicon ridge waveguide and phononic crystal slab, allowing the independent control on the confined optical and acoustic modes. The maximal small-signal Stokes gain of 0.9 dB in a 1.085 cm-long waveguide was achieved. The previously mentioned suspended waveguides inherently incorporate periodic support structures, which result in the loss of acoustic waves. Meanwhile, the material property of the photoelastic coefficient makes it challenging to observe backward SBS in silicon waveguides. Drawing inspiration from the optical antiresonance observed in hollow-core fibers and the acoustic antiresonance in cylindrical waveguides, Lei et al. propose suspended antiresonant acoustic waveguides (SARAW) with superior confinement and high selectivity of acoustic modes (Figure 10f) [171]. These waveguides offer enhanced confinement and selective mode confinement for acoustic waves and are capable of supporting both forward and backward SBS on the SOI platform. In the case of forward SBS, a centimeter-scale SARAW was shown to provide a substantial net gain surpassing 6.4 dB. For backward SBS, a remarkable Brillouin frequency shift of 27.6 GHz and a mechanical *Q* factor reaching up to 1960 were achieved. In the conventional forward SBS processes, both Stokes and anti-Stokes scattering events are facilitated by the same phonon mode. This leads to symmetric light scattering into numerous subsequent blue- and red-shifted orders, which inherently restricts the magnitude of energy transfer. Kittlaus et al. employed a multimode optomechanical waveguide (Figure 10g) [161] to generate stimulated intermodal Brillouin scattering (SIMS) in the SOI platform for the first time. This innovative system achieves decoupling of the Stokes and anti-Stokes processes by exploiting multimode dispersion to break symmetry. By leveraging this interaction, they successfully demonstrated single-sideband optical amplification and unidirectional Brillouin energy transfer in silicon. Strong Brillouin coupling enables single-sideband small-signal gain of 3.5 dB, corresponding to net on-chip amplification of 2.3 dB in this low-propagation-loss system. On this basis, Otterstrom et al. significantly enhance the Brillouin amplification process by harnessing an intermodal Brillouin interaction within a multi-spatial-mode silicon racetrack resonator (Figure 11h) [162]. They achieved net Brillouin amplification over 20 dB in silicon. Furthermore, this same system operated as a unidirectional amplifier, providing an optical nonreciprocal transmission ratio over 28 dB without insertion loss in an all-silicon platform.

While the aforementioned suspended waveguide structures can achieve Brillouin gain coefficients at the order of hundreds to thousands m^−1^ W^−1^, they still face some challenges. First, the fabrication of suspended waveguides requires high precision and limits the robustness of the devices. Second, the transmission loss of suspended silicon waveguides is typically around 1 dB/cm, which hinders large-scale circuit integration. Recently, SBS based on nonsuspended waveguide structures has been realized on the SOI platform [157], [158], [178]. Zhang et al. proposed a new optomechanical confinement approach to tightly confine photos and phonons in nonsuspended silicon waveguides by using subwavelength waveguide structures (Figure 10i) [157]. Ye et al. observed the SBS response in a nonsuspended ultralow-loss thick-SOI waveguide platform (Figure 10j) [158]. Benefit from the exceptionally low transmission loss of 2.7 dB/m and the expansive mode field area, which mitigates nonlinear loss, they achieved Brillouin gain coefficients of 2.5 m^−1 ^W^−1^ and 1.9 m^−1 ^W^−1^ at 37.6 GHz for the rib and strip waveguides, respectively.

### 5.2 Applications

Progress in Brillouin-active waveguides on the SOI platform has significantly boosted on-chip Brillouin-based applications. Among these, the silicon-based Brillouin laser stands out as a significant application, which offers powerful and flexible dynamics as the basis for mode-locked lasers [179], microwave oscillators [78], [104], [180], and optical gyroscopes [86] in a variety of optical systems. While various suspended silicon waveguide structures have successfully demonstrated significant Brillouin gain and net amplification, innovative strategies are essential to effectively convert these Brillouin interactions into functional silicon laser oscillators. Otterstrom et al. fabricated a 4.6 cm-long racetrack resonator cavity and utilized SIMS to achieve a silicon-based Brillouin laser with a linewidth of 20 kHz and a threshold of 10 mW (Figure 11a) [51]. Furthermore, they demonstrate that this silicon-based Brillouin laser enters a dynamic regime where optical self-oscillation results in the narrowing of the phonon linewidth.

Additionally, Brillouin-based acousto-optic interactions offer a bridge between microwave and optical signal processing, combining the broad bandwidth provided by photonics with the fine spectral resolution offered by acoustics. Consequently, leveraging SBS in silicon photonic chips is of significant interest in the emerging field of integrated MWP technology. MWP filters are of particular interest, serving as essential building blocks for all microwave systems. Casas-Bedoya et al. demonstrated a tunable narrowband MWP filter based on SBS in a silicon waveguide (Figure 11b) [173]. They managed to create a MWPNF with a 48-dB suppression ratio, a 98-MHz bandwidth, and a 6-GHz frequency tuning range using on-chip SBS gain of only 1 dB. Subsequently, Gertler et al. demonstrate all-silicon MWPNFs featuring a rejection ratio of 57 dB, a bandwidth of 2.7 MHz, and a frequency tuning range up to 6 GHz. This superior performance was made possible by harnessing optomechanical interactions to access long-lived phonons, significantly extending the coherence times available in silicon (Figure 11c) [174]. Moreover, microwave phase shifter elements are key modules for microwave systems. The ideal integrated microwave photonic phase shifter is capable of delivering a continuous, adjustable 360° phase shift across a wide bandwidth, characterized by minimal insertion loss and low amplitude fluctuation, all within a compact, power-efficient chip-scale design. A silicon-based Brillouin phase shifter is an attractive option. McKay et al. demonstrated a 360° broadband Brillouin-based RF phase shifter in a suspended silicon waveguide with a bandwidth of 15 GHz by using a phase enhancement method induced by RF interference (Figure 11d) [175]. Furthermore, microwave signal generation based on Brillouin scattering also plays a vital role in MWP technology. The intrinsic narrow linewidth of the SBS gain resonance makes it an attractive gain medium for generating optical and RF signals, negating the necessity for doped materials. Owing to recent progress in on-chip SBS lasers on the SOI platform, the prospects for high-performance SBS-based microwave synthesizers are particularly promising. Brillouin scattering process induced acousto-optical coupling have also been used to convert signals between optical and acoustic domains, enabling new chip-based microwave photonic functionalities such as microwave measurement. Zhou et al. developed a traveling wave electro-optomechanical system through an electrically interfaced Brillouin-active waveguide in silicon photonics (Figure 11e) [176]. Leveraging the enhancement of electro-optomechanical coupling through acoustic resonance, they achieved a bidirectional optical-to-microwave conversion with a quantum efficiency as high as 54.16 dB.

Nonreciprocal devices, specifically optical isolators, circulators, and gyrators, are essential in all photonics systems for directing light flow and protecting optical components from backscattered light. Despite significant advancements in modern integrated photonics, integrated optical nonreciprocal devices have not yet been fully realized. Brillouin scattering offers a promising solution for breaking the time-reversal symmetry in the medium, thereby achieving magnet-free nonreciprocal transmission. SBS-based isolators possess a broad bandwidth and are compatible with integrated technologies [45], [177], [181]. Kittlaus et al. demonstrated nonreciprocal single-sideband modulation and mode conversion in an integrated silicon photonic platform through linear interband Brillouin scattering (Figure 11f) [45]. This system achieved a large operational bandwidth over 125 GHz and an unprecedented nonreciprocal transmission ratio up to 38 dB between forward and backward propagating optical waves. Then, Zhou et al. introduced an innovative approach to nonreciprocal dissipation engineering by harnessing efficient interband Brillouin scattering, facilitated through electrically transduced traveling-wave phonons within a multimode optomechanical silicon waveguide (Figure 11g) [177]. Leveraging a nonreciprocal dissipation channel, they developed a frequency-neutral SBS-based isolator that boasts a low loss of less than 1 dB, a high nonreciprocal transmission ratio exceeding 14 dB, and a broad operating bandwidth surpassing 95 GHz.

## 6 Other materials

### 6.1 Silicon nitride

**Figure 12:**
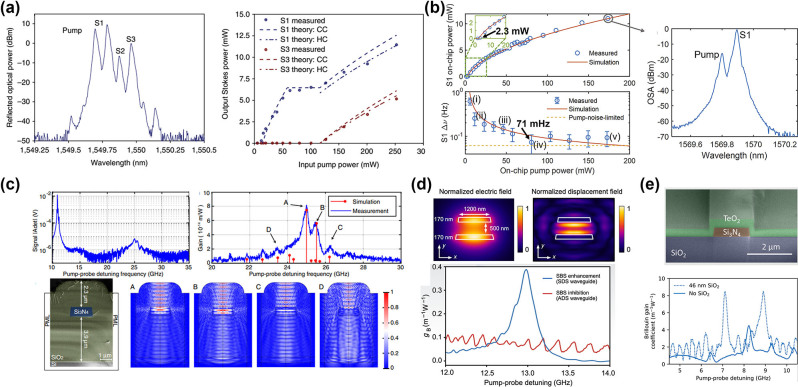
Si_3_N_4_-based SBS and its applications. (a) Brillouin laser with sub-hertz intrinsic linewidth. Reproduced with permission [52]. Copyright 2019, Springer Nature. (b) Sub-100-mHz fundamental linewidth [110]. Copyright 2024, Optica Publishing Group. (c) SBS in thick Si_3_N_4_ waveguides (with 800-nm thickness) [25]. (d) SBS in multilayer Si_3_N_4_ waveguides [196]. (e) SBS in Si_3_N_4_–TeO_2_ hybrid waveguides [113].

Silicon nitride (Si_3_N_4_) has emerged as a promising low-loss and multifunctional integration platform, finding applications in a spectrum of cutting-edge technologies such as lasers [182], frequency combs [183], [184], [185], microwave photonics [186], [187], [188], isolators [189], [190], and integrated on-chip amplifiers [191]. Within Si_3_N_4_ waveguides, nonlinear phenomena such as second-harmonic and third-harmonic generation [192], [193], along with stimulated Raman scattering [194], have garnered significant research attention. In contrast, the study of Brillouin scattering in Si_3_N_4_ is a relatively recent development in the field. In 2019, Gundavarapu et al. demonstrated the SBS effect in thin Si_3_N_4_ waveguides (with 40-nm thickness), with a Brillouin gain coefficient of 0.1 m^−1^ W^−1^ (Figure 12a) [52]. Furthermore, they achieved a Brillouin laser with sub-hertz intrinsic linewidth based on ultrahigh-*Q* microring resonators, which had a threshold of 14.6 mW, and showcased its applications in optical gyroscopes and low-noise photonic oscillators. By further reducing the waveguide loss to 0.034 dB/m, the threshold for Brillouin lasing was lowered to 380 µW in 80 nm-thick Si_3_N_4_ waveguides [108]. Recently, based on a photonic molecule coupled resonator design (Figure 12b) [110], Liu et al. achieved Brillouin lasing in Si_3_N_4_ waveguides with a fundamental linewidth below 100 mHz and an output power over 10 mW in the C band. Additionally, visible light photonic integrated Brillouin laser with emission at 674 nm, a 14.7-mW lasing threshold has been demonstrated in Si_3_N_4_ waveguides [107]. The aforementioned work was all carried out on a thin-Si_3_N_4_ platform. The SBS interaction occurred within the silica cladding. In 2020, Guger et al. observed backward SBS in thick Si_3_N_4_ waveguides (with 800-nm thickness) for the first time (Figure 12c) [25]. The reported intrinsic Brillouin gain coefficient at 25 GHz is estimated to be 0.07 m^−1^ W^−1^. The aforementioned demonstrations, whether involving thin or thick Si_3_N_4_ waveguides, were all troubled by acoustic leakage from the silicon nitride core to the surrounding silica cladding. This leakage prevented the enhancement of SBS in nanophotonic waveguides, leading to lower SBS gain. In 2022, Botter et al. realized a large backward Brillouin gain coefficient of 0.53 m^−1^ W^−1^ in multilayer Si_3_N_4_ waveguides (Figure 12d). Furthermore, they used the enhanced SBS gain to demonstrate a MWPNF with a high rejection ratio of 66 dB. Recently, Botter et al. measured a Brillouin gain coefficient of f 8.5 m^−1^ W^−1^ in a scalable Brillouin platform combining low-loss Si_3_N_4_ and tellurium oxide (TeO_2_) (Figure 12e) [195]. Following up on the same platform, Klaver et al. significantly boosted the Brillouin gain coefficient to 81 m^−1^ W^−1^ through geometric optimization and advanced cladding engineering [113]. Leveraging this enhancement, they successfully developed a Si_3_N_4_ Brillouin amplifier exhibiting net gain of 5 dB, a Brillouin laser with an intrinsic linewidth of just 7 Hz, and a widely tunable MWPNF featuring an ultra-narrow bandwidth of 2.2 MHz.

### 6.2 Thin-film lithium niobate

**Figure 13:**
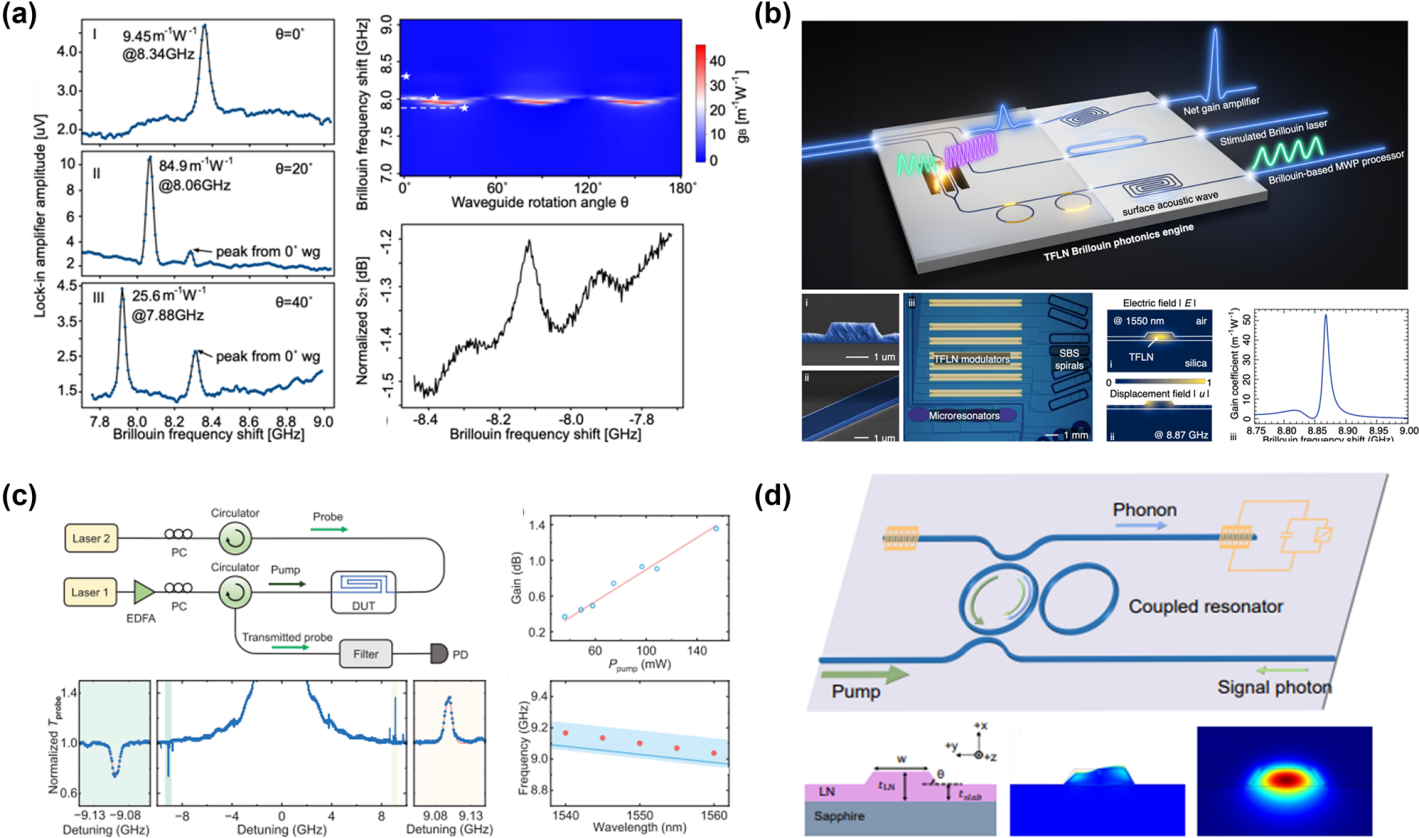
SBS in TFLN waveguides and its applications (a) SBS in TFLN waveguides, facilitated by SAW [203]. (b) Brillouin photonics engine in TFLN waveguides [26]. (c) SBS based on TFLN-on-sapphire platform. Reproduced with permission [205]. Copyright 2023, Science China Press. (d) Microwave-to-optical quantum transduction based on cavity-enhanced Brillouin interaction [206].

Lithium niobate (LN) is a traditional optical material with excellent performance, often hailed as silicon of photonics. In recent years, the development of thin-film lithium niobate (TFLN) fabrication technology has garnered unprecedented attention. TFLN waveguides are renowned for their low loss, scalability, and versatility [197], [198], achieving unprecedented performance and functionality in modulators [199], optical frequency combs [200], and quantum optics [201]. Recently, theoretical studies have forecast a significant SBS effect in TFLN waveguides [202]. Subsequently, Ye et al. and Rodrigues et al. independently and nearly simultaneously observed the backward SBS signals in TFLN waveguides (Figure 13a) [203], [204], facilitated by surface acoustic waves (SAW). Both teams reported achieving a Brillouin gain coefficient that surpassed 80 m^−1^ W^−1^. Building on this foundation, Ye et al. successfully implemented a Brillouin laser based on TFLN waveguides as well as Brillouin-based MWPNFs (Figure 13b) [26]. The aforementioned studies all depend on SAWs to amplify Brillouin interactions, whereas phonons, unlike photons, are not confined within the TFLN waveguides. Recently, Yang et al. achieved a simultaneous tight confinement of both phononic and photonic modes by using a TFLN-on-sapphire chip (Figure 13c) [205]. Subsequently, they introduced a novel scheme for efficient and low-noise conversion of microwave-to-optical quantum signals, leveraging cavity-enhanced Brillouin interaction between telecom photons and 10 GHz phonons on TFLN-on-sapphire platform (Figure 13d) [206].

### 6.3 III–V materials (AlGaAs, AlGaN, and AlN)

**Figure 14:**
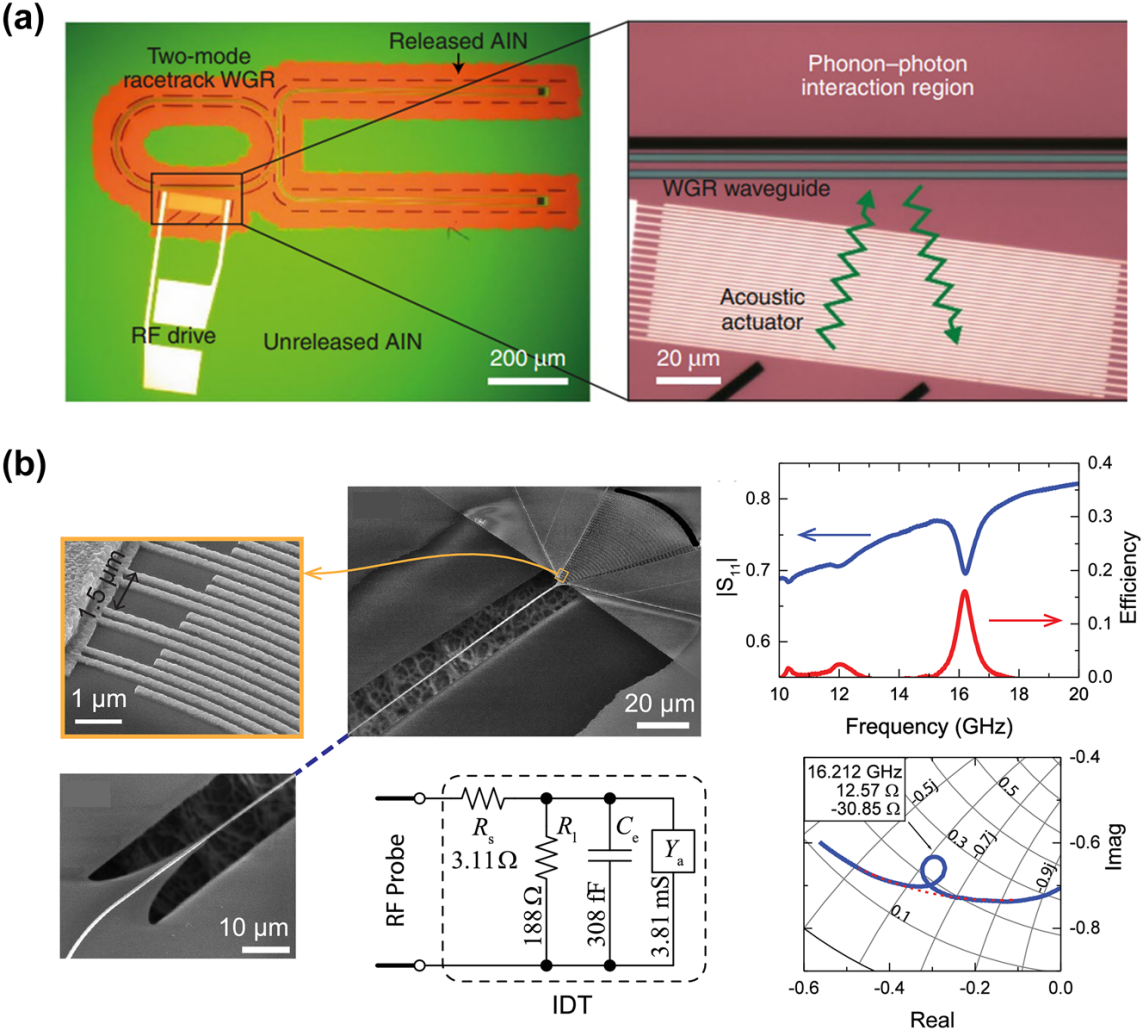
SBS based on III–V waveguide platform. (a) AlN racetrack resonator used with acoustic modulation. Reproduced with permission [210]. Copyright 2018, Springer Nature. (b) Electromechanically excited Brillouin scattering in integrated optomechanical AlN waveguides [211].

III–V materials exhibit exceptionally large nonlinear coefficients, which are significantly greater than those found in the dielectric materials previously mentioned. Moreover, these materials boast higher refractive indices, enabling the attainment of a pronounced index contrast [207]. This leads to a reduced modal cross-sectional area, resulting in a higher intensity. The combination of these attributes is highly desirable, as it diminishes the threshold power required to initiate nonlinear optical phenomena within semiconductor-based platforms. Recently, researchers have shown a keen interest in the Brillouin interaction within III–V materials. AlN is a novel III–V material with a wide transparent window from 200 nm and an appropriate refractive index to confine the light [208]. It is an excellent material for optomechanical interactions since it has a very large acoustic lifetime and because acoustic waves can be directly excited electronically [209]. In 2018, Sohn et al. successfully observed Brillouin scattering from induced acoustic waves in suspended AlN waveguides (Figure 14a) [210]. Utilizing this method, they crafted a nonreciprocal modulator, functioning as a frequency-shifting isolator, through the mechanism of indirect interband scattering. Subsequently, Liu et al. showcased the generation of Brillouin scattering through electro-mechanical excitation in integrated optomechanical AlN waveguides (Figure 14b) [211]. They successfully excited acoustic phonons at a frequency of 16 GHz using nanofabricated electromechanical transducers, which then scattered counterpropagating photons within the waveguide into a single anti-Stokes sideband, demonstrating the potential for advanced photonic applications. Recently, Li et al. proposed a partially suspended AlN waveguide operating at 450 nm, achieving a SBS gain of 1,311 m^−1^ W^−1^, where the Brillouin scattering is optically excited [212]. In addition to AlN, SBS has also been observed in AlGaAs [213] and AlGaN [214].

### 6.4 Fluoride (CaF_2_, LiF, BaF_2_, SrF_2_, and MgF_2_)

**Figure 15:**
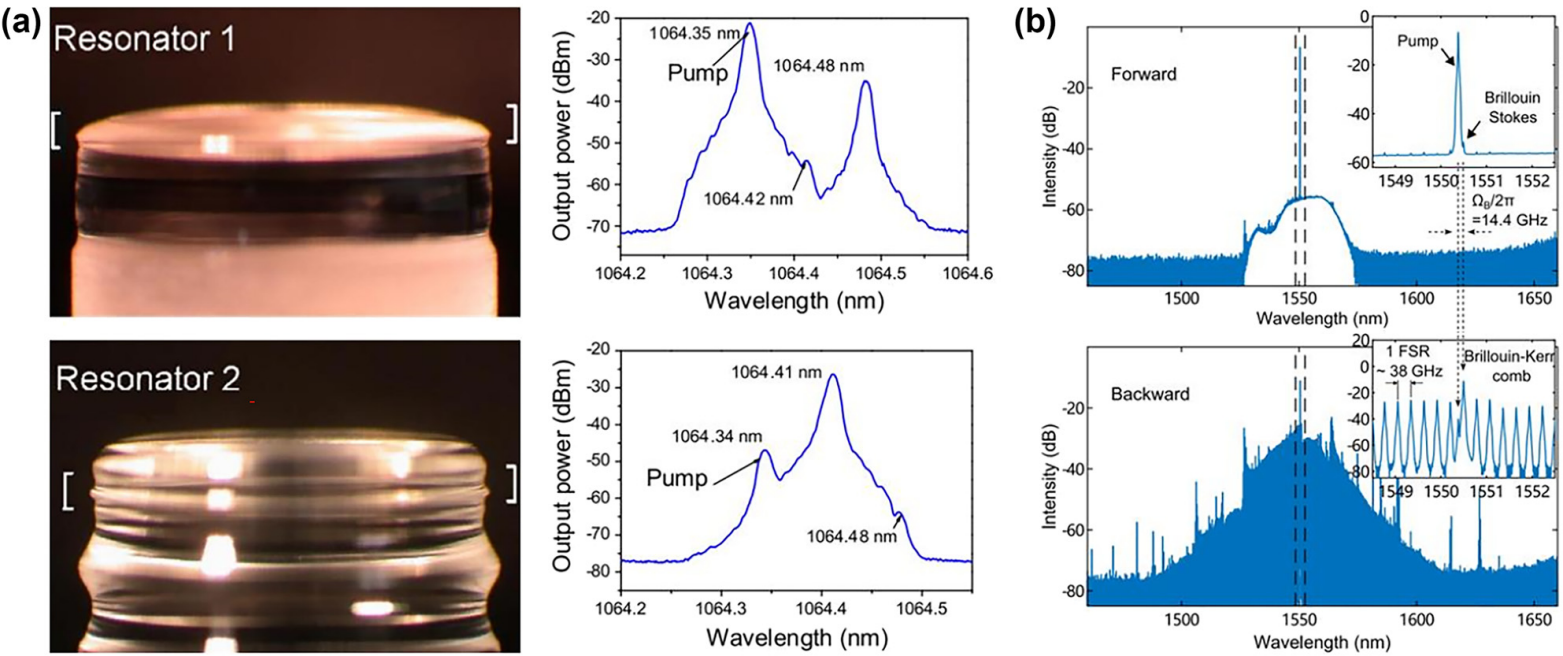
SBS in fluoride resonators. (a) Stimulated Brillouin lasing in CaF_2_ WGM resonators. Reproduced with permission [54]. Copyright 2009, American Physical Society. (b) Brillouin–Kerr frequency comb generation in a MgF_2_ WGM resonator [217].

Fluoride crystalline materials commonly possess high nonlinear coefficients, have transparent windows that extend from the ultraviolet to the mid-infrared, and exhibit flat anomalous group velocity dispersion ranging from the visible to the mid-infrared band. Due to the low absorption loss of these materials, as well as the large size and low scattering loss of the crystalline resonators, the *Q* factor of fluoride crystalline resonators is typically higher than 10^9^. In 2009, Grudinin et al. initially demonstrated the forward and backward Brillouin scattering in ultrahigh-*Q* calcium fluoride (CaF_2_) crystalline resonators, where the FSR was matched to the Brillouin frequency shift (Figure 15a) [54]. Under the pumping wavelength of 1,064 nm, a Brillouin laser with a threshold of 3 μW was attained. In the following years, researchers had reported SBS in crystalline resonators composed of different fluoride materials. Diallo et al. accomplished SBS successively in lithium fluoride (LiF) [215], barium fluoride (BaF_2_) [105], [216], and strontium fluoride (SrF_2_) [216]. In addition, the authors observed Kerr–Brillouin interaction in BaF_2_ and SrF_2_ resonators. Recently, Lin et al. first reported the backward stimulated Brillouin in Z-cut magnesium fluoride (MgF_2_) resonators and demonstrated the generation of Brillouin–Kerr frequency combs, as shown in Figure 15b [217], [218]. Later, Xu et al. observed the same phenomenon [109]. Besides, the authors reported demonstrating the excitation of Brillouin–Kerr optical frequency comb based on CaF_2_ crystalline resonators [219], [220].

## 7 Conclusions

In this review, we have summarized the research progress and applications of SBS across various material platforms, with a primary focus on micro/nanoscale waveguides and resonators. Although significant progress has been made, there are still major challenges to be addressed for this exciting technology to have a widespread impact on real-world applications. For silica and fluoride, although microcavities with ultrahigh *Q*-factors have been achieved, enabling the realization of Brillouin lasers with sub-Hz intrinsic linewidths that can meet almost all application requirements, including atomic clocks, the trend for future development lies in achieving full on-chip integration. Moreover, there is a direct trade-off between the *Q*-factor and the laser output power, and optimizing this trade-off to achieve the best performance is a key issue that needs to be addressed. For silicon, the primary challenge is to confine both photons and phonons within nonsuspended waveguide structures while preserving a high Brillouin gain coefficient. Although various solutions have been put forth, the enhancement of the Brillouin gain coefficient continues to be a crucial goal. Chalcogenide glasses require ongoing exploration of new compositions to significantly boost the laser damage threshold. Concurrently, it is imperative to study the feasibility of integrating these materials with other predominant photonic integration platforms [221], [222]. For silicon nitride and lithium niobate, limitations in material properties make it difficult to further increase the Brillouin gain coefficient; hence, heterogeneous integration is also a promising direction for development. For III–V materials, their broad transparency window makes them highly suitable for SBS research in the visible spectrum. Additionally, these materials exhibit strong acousto-optic coupling and large electromechanical transduction efficiency, which can be utilized to achieve nonreciprocal devices through acousto-optics and Brillouin phase matching.

We are convinced of the escalating importance of SBS within photonic applications. Investigating Brillouin scattering within micro- and nanoscale photonic structures is essential for propelling the development of practical applications as well as providing new opportunities for fundamental studies.
